# Supplementary material for: Tailoring interventions to suit self-reported format preference does not decrease vaccine hesitancy
Source: PLoS One. 2023 Mar 21;18(3):e0283030. doi: 10.1371/journal.pone.0283030 (PMC10030039; doi:10.1371/journal.pone.0283030)
Supplement: S1 File — (PDF) [file pone.0283030.s001.pdf]

Simulation: Tailoring Interventions to Suit Self-Reported Format Preference Does Not Decrease Vaccine Hesitancy

Otto Mäki

Contents

Required packages **1**

Create and test the power function **1**

Summary **4**

Required packages

```
library(tidyverse)
library(broom)
```

Create and test the power function

To test different configurations of effect sizes vs. sample sizes, we can create our own custom function which takes `effect` and `sample size` as inputs and produces power estimates as outputs. In this case, we create the variable *format preference* (fp) with a mean of 0 and a standard deviation of 1, the variable *vaccination intention* (v_intention) that is dependent on format preference in groups *stat* and *anec*, and has a random variance with a mean of 0 and a standard deviation of 10. To test the statistical power of our analyses, we run the linear model we plan to run in the study, in this case `lm(v_intention ~ fp*group)`, and we pull out the p value for the interaction between format preference and the group anec and then between format preference and group stat. When we run the code for example 500 times we get the statistical power by looking at the proportion of significant results against non-significant results. Since we know for a fact that the variable `v_intention` is dependent on the variable `fp` as we have coded it, we can test how many participants will be needed to reliably show that the effect size is statistically significant.

```
my_power_function_anec <- function(effect, sample_size) {

# Statistically significant results for the anecdotal group
  sig_results_anec <- c()

  for(i in 1:500) {
  #Create the data
    group_stat <- tibble(fp = rnorm(sample_size, mean = 0, sd= 1)) %>%
      mutate(v_intention = -effect*fp + rnorm(sample_size, mean = 0, sd = 10)) %>%
      mutate(group = "stat")

    group_anec <- tibble(fp = rnorm(sample_size, mean = 0, sd= 1)) %>%
      mutate(v_intention = effect*fp + rnorm(sample_size, mean = 0, sd = 10)) %>%
      mutate(group = "anec")
```

```
    group_cont <- tibble(fp = rnorm(sample_size, mean = 0, sd= 1)) %>%
      mutate(v_intention = rnorm(sample_size, mean = 0, sd = 10)) %>%
      mutate(group = "cont")

    tib3 <- rbind(group_stat, group_anec, group_cont)

    tib3 <- tib3 %>% mutate(group = fct_relevel(group, "cont"))

    # Run the analysis
    m3 <- lm(v_intention ~ fp*group, data = tib3)

    # Get the results
    sig_results_anec[i] <- tidy(m3)$p.value[5] <= .05
  }

  sig_results_anec %>%
    mean() %>%
    return()
}
```

To test our function, we now fill in the effect and sample sizes we wish to test. In this case we want to test whether sample sizes of 30, 50, 100, 200, and 300 per group are large enough to detect a small (3 point) change in v_intention.

```
set.seed(1996)
my_power_function_anec(3, 30)
```

```
## [1] 0.226
```

```
set.seed(1996)
my_power_function_anec(3, 50)
```

```
## [1] 0.288
```

```
set.seed(1996)
my_power_function_anec(3, 100)
```

```
## [1] 0.584
```

```
set.seed(1996)
my_power_function_anec(3, 200)
```

```
## [1] 0.85
```

```
set.seed(1996)
my_power_function_anec(3, 300)
```

```
## [1] 0.956
```

With a total sample size of `600` (200 per group) we get an acceptable statistical power of `0.85` for the interaction between format preference and the anecdotal group with control group as baseline. Lets check the statistical power for the interaction between format preference and the stat group.

```
my_power_function_stat <- function(effect, sample_size) {

# Statistically significant results for the statistical group
  sig_results_stat <- c()
```

```r
  for(i in 1:500) {
  #Recreate the data
    group_stat <- tibble(fp = rnorm(sample_size, mean = 0, sd= 1)) %>%
      mutate(v_intention = -effect*fp + rnorm(sample_size, mean = 0, sd = 10)) %>%
      mutate(group = "stat")

    group_anec <- tibble(fp = rnorm(sample_size, mean = 0, sd= 1)) %>%
      mutate(v_intention = effect*fp + rnorm(sample_size, mean = 0, sd = 10)) %>%
      mutate(group = "anec")

    group_cont <- tibble(fp = rnorm(sample_size, mean = 0, sd= 1)) %>%
      mutate(v_intention = rnorm(sample_size, mean = 0, sd = 10)) %>%
      mutate(group = "cont")

    tib4 <- rbind(group_stat, group_anec, group_cont)

    tib4 <- tib4 %>% mutate(group = fct_relevel(group, "cont"))

    # Run the analysis
    m3 <- lm(v_intention ~ fp*group, data = tib4)

    # Get the results
    sig_results_stat[i] <- tidy(m3)$p.value[6] <= .05
  }

  sig_results_stat %>%
    mean() %>%
    return()
}
```

```r
set.seed(1996)
my_power_function_stat(3, 30)
```

```
## [1] 0.192
```

```r
set.seed(1996)
my_power_function_stat(3, 50)
```

```
## [1] 0.336
```

```r
set.seed(1996)
my_power_function_stat(3, 100)
```

```
## [1] 0.542
```

```r
set.seed(1996)
my_power_function_stat(3, 200)
```

```
## [1] 0.886
```

```r
set.seed(1996)
my_power_function_stat(3, 300)
```

```
## [1] 0.948
```

With a total sample size of `600` (200 per group) we get a statistical power of `0.886` for the interaction between format preference and the statistical group.

Summary

Taken together, we get a statistical power for the interaction between format preference and the anecdotal and statistical groups of around `0.87` ($(0.85 + 0.886)/2 = 0.868$) with a small effect size and a total sample of `600`.
