# Supplementary material for: Tailoring interventions to suit self-reported format preference does not decrease vaccine hesitancy
Source: PLoS One. 2023 Mar 21;18(3):e0283030. doi: 10.1371/journal.pone.0283030 (PMC10030039; doi:10.1371/journal.pone.0283030)
Supplement: S2 File — (DOCX) [file pone.0283030.s002.docx]

**S2 File. Supporting information for the COVID-19 and influenza experiments.**

| **S1 Table** | | | | |
| --- | --- | --- | --- | --- |
| *Sample Demographics – COVID-19 and Influenza Experiments* | | | | |
| Variable | COVID-19 Experiment | | Influenza Experiment | |
|  | *n* | % | *n* | % |
| Age |  |  |  |  |
| 18 – 29 | 49 | 8.8 | 69 | 12.8 |
| 30 – 39 | 104 | 18.6 | 126 | 23.3 |
| 40 – 49 | 135 | 24.2 | 115 | 21.3 |
| 50 – 59 | 106 | 19.0 | 73 | 13.5 |
| 60 – 69 | 48 | 8.6 | 74 | 13.7 |
| 70 – 79 | 19 | 3.4 | 16 | 3.0 |
| 80 – 89 | 1 | 0.2 | 1 | 0.2 |
| Missing | 97 | 17.4 | 66 | 12.2 |
| Gender |  |  |  |  |
| Male | 99 | 17.7 | 118 | 21.9 |
| Female | 423 | 75.7 | 392 | 72.6 |
| Other | 6 | 1.1 | 7 | 1.3 |
| Did not want to report | 27 | 4.8 | 19 | 3.5 |
| Missing | 4 | 0.7 | 4 | 0.7 |
| Education |  |  |  |  |
| Lower | 225 | 40.3 | 204 | 37.8 |
| Higher | 314 | 56.2 | 321 | 59.4 |
| Other | 17 | 3.0 | 14 | 2.6 |
| Missing | 3 | 0.5 | 1 | 0.2 |
| Region |  |  |  |  |
| Uusimaa | 181 | 32.4 | 184 | 34.1 |
| Varsinais-Suomi | 71 | 12.7 | 78 | 14.4 |
| Pirkanmaa | 33 | 5.9 | 47 | 8.7 |
| Other | 268 | 47.9 | 227 | 42.0 |
| Missing | 6 | 1.1 | 4 | 0.7 |
| Worked as a healthcare worker |  |  |  |  |
| Yes | 213 | 38.1 | 114 | 21.1 |
| No | 343 | 61.4 | 424 | 78.5 |
| Missing | 3 | 0.5 | 2 | 0.4 |
| N | 559 | 100% | 540 | 100% |

| **S1 Fig** | | | | | | | |
| --- | --- | --- | --- | --- | --- | --- | --- |
| *Overview of Procedure – COVID-19 and Influenza Vaccine Hesitancy Interventions* | | | | | | | |
|  | | | | | | | |
| Informed consent | | | | | | | |
|  | | | |  | | | |
| Vaccinations and vaccination experiences | | | | | | | |
| Trust in health authorities | | | | | | | |
| Vaccine confidence | | | | | | | |
| Conspiracy mentality | | | | | | | |
|  | | |  | |  | | |
| **COVID-19 Group**  (n = 559) | | |  | | **Influenza Group**  (n = 540) | | |
|  |  |  |  | |  |  |  |
|  | | | 1^st^ Intention > 80% | |  | | |
| Intention to take a seasonal COVID-19 vaccine | | |  | | Intention to take next influenza vaccine | | |
|  | | |  | |  | | |
| Intention ≤ 80% | | | 2^nd^ Intention > 80% | | Intention ≤ 80% | | |
|  | | |  | |  | | |
| *Format preference | | |  | | *Format preference | | |
|  | | |  | |  | | |
| Intention to take a third COVID-19 vaccine dose | | |  | | - | | |
|  | | |  | |  | | |
| COVID-19 vaccination easiness | | |  | | Influenza vaccination easiness | | |
|  | | |  | |  | | |
| COVID-19 vaccine safety | | |  | | Influenza vaccine safety | | |
|  | | |  | |  | | |
| COVID-19 vaccine efficacy | | |  | | Influenza vaccine efficacy | | |
|  | | |  | |  | | |
| COVID-19 threat | | |  | | Influenza threat | | |
|  | | |  | |  | | |
| COVID-19 vaccination altruism | | |  | | Influenza vaccination altruism | | |
|  | | |  | |  | | |
| Intervention | | |  | | Intervention | | |
| Statistical | Anecdotal | Control |  | | Statistical | Anecdotal | Control |
|  | | |  | |  | | |
| Emotional response | | |  | | Emotional response | | |
|  | | |  | |  | | |
| Intention to take a seasonal COVID-19 vaccine | | |  | | Intention to take next influenza vaccine | | |
|  | | |  | |  | | |
| Intention to take a third COVID-19 vaccine dose | | |  | | - | | |
|  | | |  | |  | | |
| Intervention effect | | |  | | Intervention effect | | |
|  | | |  | |  | | |
| Message relevance | | |  | | Message relevance | | |
|  | | |  | |  | | |
| Message helpfulness | | |  | | Message helpfulness | | |
|  | | |  | |  | | |
| COVID-19 vaccination easiness | | |  | | Influenza vaccination easiness | | |
|  | | |  | |  | | |
| COVID-19 vaccine safety | | |  | | Influenza vaccine safety | | |
|  | | |  | |  | | |
| COVID-19 vaccine efficacy | | |  | | Influenza vaccine efficacy | | |
|  | | |  | |  | | |
| COVID-19 threat | | |  | | Influenza threat | | |
|  | | |  | |  | | |
| COVID-19 vaccination altruism | | |  | | Influenza vaccination altruism | | |
|  | | |  | |  | | |
| Demographic information | | |  | | Demographic information | | |
|  | | |  | |  | | |
| *Format preference | | |  | | *Format preference | | |
|  | | |  | |  | | |
| End page | | | | | | | |
| *Note.* Steps color coded as grey were shared across both groups. | | | | | | | |

**S2 Fig**

*Statistical Intervention – COVID-19 ORIGINAL*

*
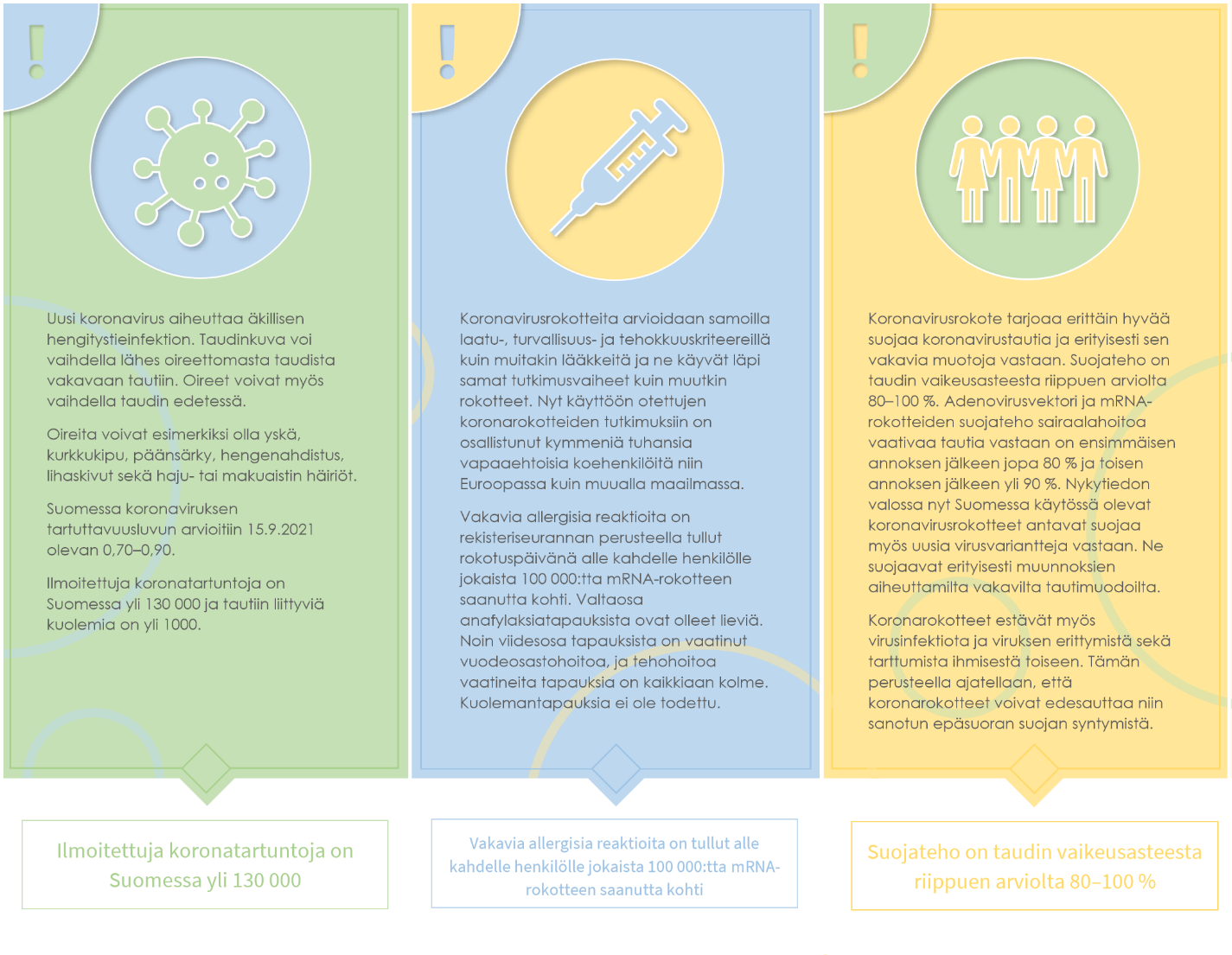
*

**S2 Table**

*Statistical Intervention – COVID-19 TRANSLATIONS*

| The new coronavirus causes a sudden respiratory infection. The clinical picture can vary from almost asymptomatic to severe disease. Symptoms may also vary as the disease progresses.  Symptoms may include cough, sore throat, headache, shortness of breath, muscle aches, and disturbances in sense of smell or taste.  In Finland, the estimated reproductive number of the coronavirus was 0.70–0.90 on 15 September 2021.  There are more than 130 000 reported cases of coronavirus infection in Finland and more than 1 000 deaths related to the disease. | Coronavirus vaccines are assessed against the same quality, safety and efficacy criteria as other medicines and go through the same research stages as other vaccines. The coronavirus vaccines now in use have involved tens of thousands of volunteers in trials in Europe and around the world.  Based on registry surveillance, the number of severe allergic reactions on the day of vaccination has been less than two for every 100 000 people who received the mRNA vaccine. The vast majority of anaphylaxis cases have been mild. About one fifth of cases have required hospitalization, and there have been three cases requiring intensive care. No deaths have been reported. | The coronavirus vaccine offers very good protection against COVID-19, especially against its severe forms. Depending on the severity of the disease, the protection level is estimated to be between 80% and 100%. The protective efficacy of adenovirus vector and mRNA vaccines against hospital-acquired disease is up to 80% after the first dose and over 90% after the second dose. In the light of current knowledge, the coronavirus vaccines now in use in Finland also provide protection against new viral variants. In particular, they protect against severe forms of disease caused by variants.  Coronavirus vaccines also prevent viral infection and virus shedding and transmission from one person to another. On this basis, it is thought that coronavirus vaccines may contribute to the so-called indirect protection. |
| --- | --- | --- |
| There are more than 130 000 reported cases of corona infection in Finland | Severe allergic reactions have occurred in fewer than two people for every 100 000 mRNA vaccine recipients | Depending on the severity of the disease, the protection level is estimated to be between 80% and 100% |

**S3 Fig**

*Anecdotal Intervention – COVID-19 ORIGINAL*

*
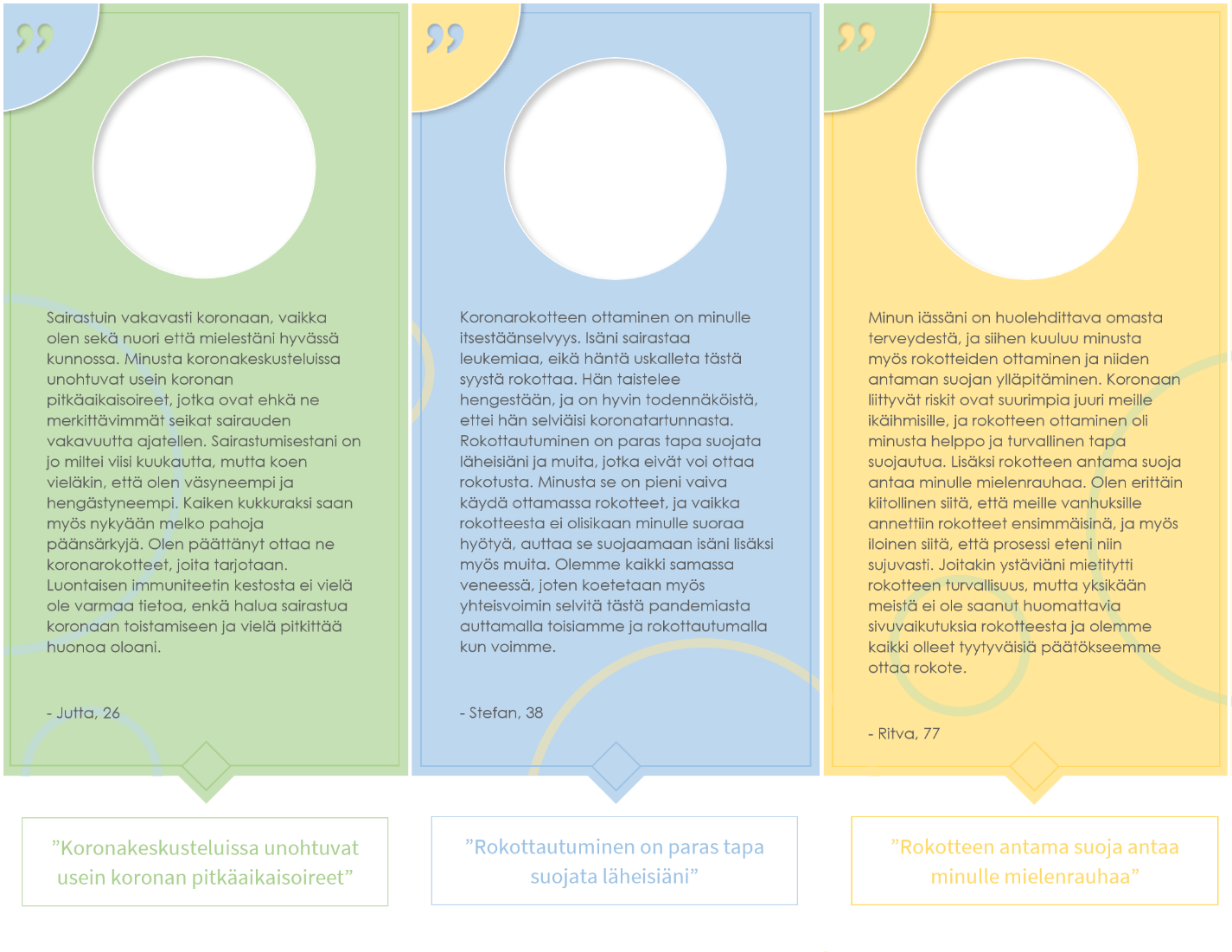
*

**S3 Table**

*Anecdotal Intervention – COVID-19 TRANSLATIONS*

| I became seriously ill with COVID-19, even though I am both young and I think I am in good shape. I think that the long-term symptoms of COVID-19 are often forgotten in discussions about the disease, which are perhaps the most important aspects of its severity. It has been almost five months since I became ill, and I still feel tired and out of breath. To make matters worse, I now get quite bad headaches. I have decided to take the COVID-19 vaccines that are being offered. The duration of natural immunity is not yet known for sure, and I do not want to get COVID-19 again and prolong my misery.  - Jutta, 26 | For me, taking the COVID-19 vaccine is a no-brainer. My father has leukemia and for this reason he is not allowed to be vaccinated. He is fighting for his life and it is very likely that he would not survive a COVID-19 infection. Getting the vaccine is the best way to protect my loved ones and others who cannot have the vaccine. I think it's a small hassle to get the vaccine, and even if it doesn't directly benefit me, it will help protect not only my father but others. We are all in the same boat, so let's also try to work together to get through this pandemic by helping each other and getting vaccinated when we can.  - Stefan, 38 | At my age, you have to take care of your own health, and I think that includes taking vaccinations and maintaining the protection they provide. The risks associated with COVID-19 disease are greatest for us, the elderly, and I felt that taking the vaccine was an easy and safe way to protect myself. In addition, the protection provided by the vaccine gives me peace of mind. I am very grateful that we elderly people were the first to be vaccinated, and I am also glad that the process went so smoothly. Some of my friends were concerned about the safety of the vaccine, but none of us have had any significant side effects and we have all been happy with our decision to take the vaccine.  - Ritva, 77 |
| --- | --- | --- |
| "The long-term symptoms of COVID-19 are often forgotten in discussions about the disease" | "Getting the vaccine is the best way to protect my loved ones" | "The protection provided by the vaccine gives me peace of mind" |

**S4 Fig**

*Statistical Intervention – Influenza ORIGINAL*


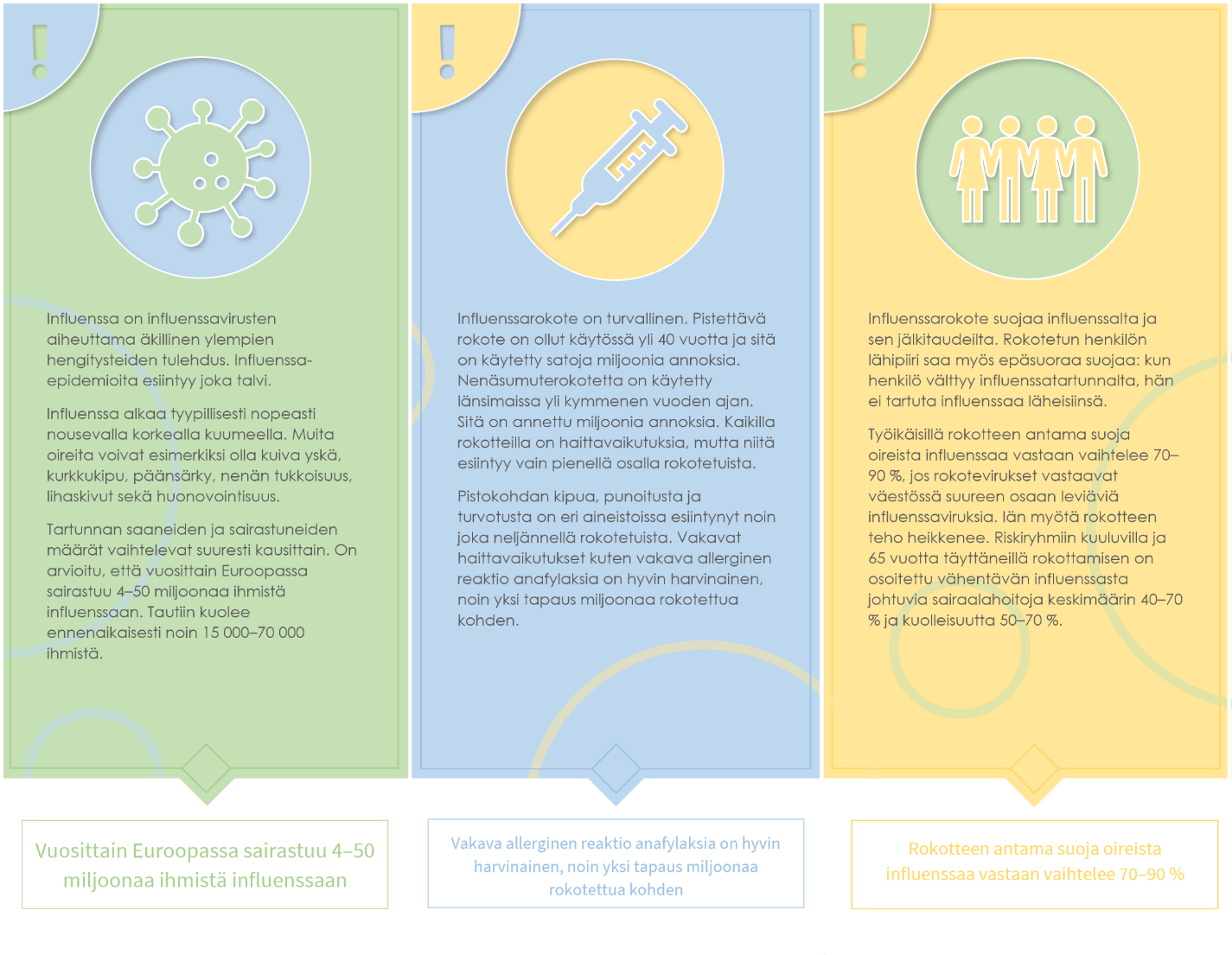


**S4 Table**

*Statistical Intervention – Influenza TRANSLATIONS*

| Influenza is a sudden inflammation of the upper respiratory tract caused by influenza viruses. Influenza epidemics occur every winter.  Influenza typically starts with a rapidly rising high fever. Other symptoms may include dry cough, sore throat, headache, nasal congestion, muscle aches, and malaise.  The numbers of people infected and affected vary greatly from season to season. It is estimated that between 4 and 50 million people in Europe contract influenza each year. Between 15 000 and 70 000 people die prematurely. | The flu vaccine is safe. The injectable vaccine has been in use for over 40 years and hundreds of millions of doses have been used. The nasal spray vaccine has been used in the West for more than a decade. Millions of doses have been given. All vaccines have side effects, but only a small proportion of those vaccinated experience them.  Pain, redness and swelling at the injection site have been reported in about one in four of those vaccinated. Serious adverse reactions such as severe allergic reaction anaphylaxis are very rare, about one case per million vaccinees. | The flu vaccine protects against influenza and its after-effects. The vaccinated person's immediate family also receives indirect protection: when a person avoids influenza infection, they do not spread the flu to their loved ones.  In working-age people, the vaccine protection against symptomatic influenza ranges from 70-90% if the vaccine viruses correspond to the influenza viruses that are circulating in the population in large numbers. The effectiveness of the vaccine declines with age. In people at risk and those aged 65 and over, vaccination has been shown to reduce influenza-related hospitalizations by an average of 40-70% and mortality by 50-70%. |
| --- | --- | --- |
| Between 4 and 50 million people in Europe contract influenza each year | Serious adverse reactions such as severe allergic reaction anaphylaxis are very rare, about one case per million vaccinees | Vaccine protection against symptomatic influenza ranges from 70-90% |

**S5 Fig**

*Anecdotal Intervention – Influenza ORIGINAL*


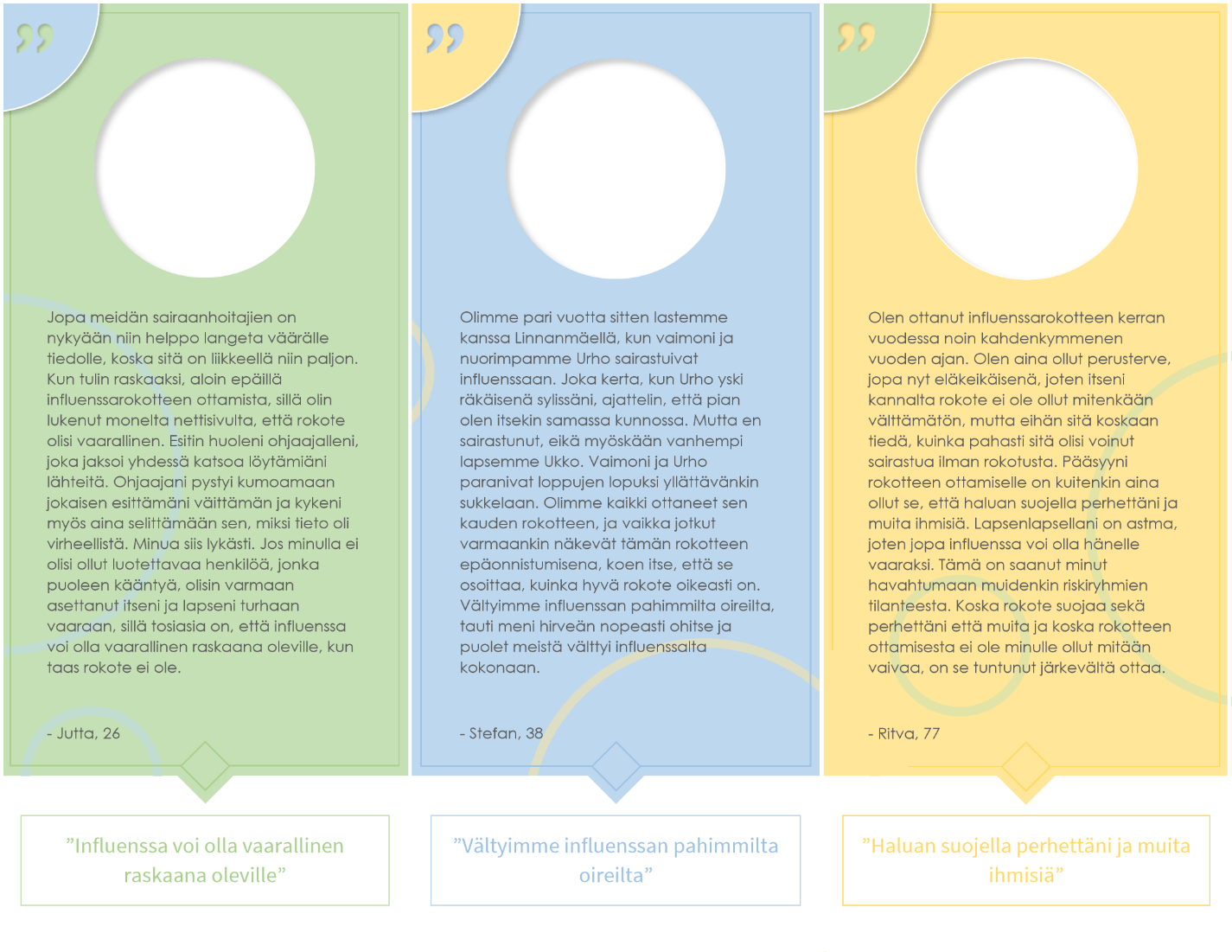


**S5 Table**

*Anecdotal Intervention – Influenza TRANSLATIONS*

| Even for us nurses today, it's so easy to fall for misinformation because there's so much of it out there. When I became pregnant, I started to have doubts about taking the flu vaccine, as I had read on many websites that it was dangerous. I voiced my concerns to my supervisor, who took the time to look at the sources I had found together. My tutor was able to refute every claim I made and was always able to explain why the information was incorrect. So I was lucky. If I had not had a reliable person to turn to, I would probably have put myself and my child in unnecessary danger, because the fact is that influenza can be dangerous for pregnant women, while the vaccine is not.  - Jutta, 26 | A couple of years ago, we were at Linnanmäki with our children when my wife and our youngest, Urho, came down with the flu. Every time Urho coughed in my lap, I thought that I would soon be in the same condition myself. But I didn't get sick, and neither did our older child, Ukko. My wife and Urho recovered surprisingly quickly in the end. We had all had the vaccine that season, and although some will probably see this vaccine as a failure, I personally feel that it shows how good the vaccine really is. We avoided the worst symptoms of the flu, the disease passed very quickly and half of us avoided the flu altogether.  - Stefan, 38 | I have had the flu vaccine once a year for about twenty years. I have always been in good health, even now in retirement, so for me the vaccine has not been necessary, but you never know how badly you could have gotten sick without it. However, my main reason for getting the vaccine has always been that I want to protect my family and other people. My granddaughter has asthma, so even the flu could be a danger to her. This has made me aware of the situation of other people at risk. Because the vaccine protects my family and others, and because it has been no trouble for me to take the vaccine, it has made sense for me to take it.  - Ritva, 77 |
| --- | --- | --- |
| "Influenza can be dangerous for pregnant women" | "We avoided the worst symptoms of the flu" | "I want to protect my family and other people" |

**S6 Fig**

*Control Material – COVID-19 and Influenza ORIGINAL*


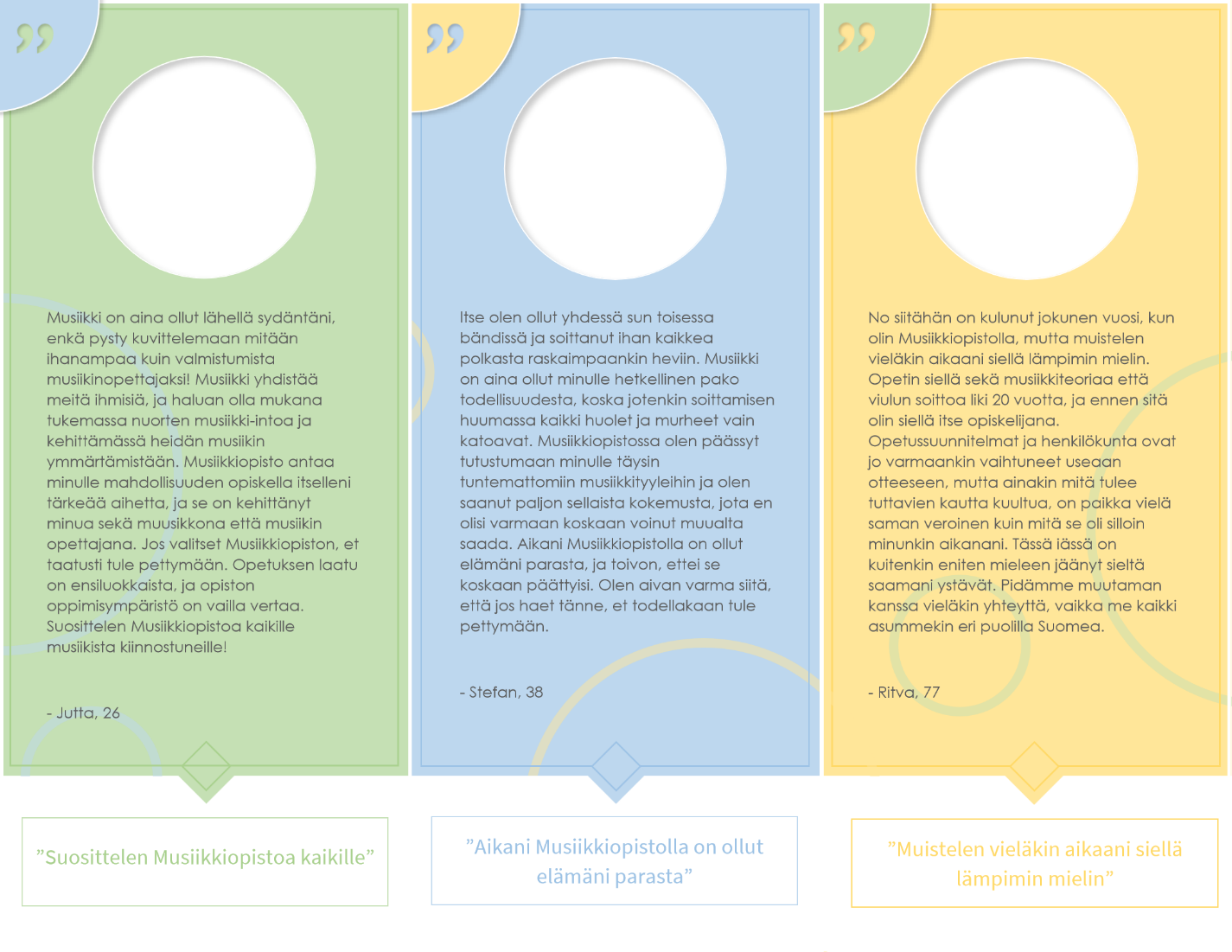


**S6 Table**

*Control Material – COVID-19 and Influenza TRANSLATIONS*

| Music has always been close to my heart, and I can't imagine anything more wonderful than graduating as a music teacher! Music brings us all together as human beings, and I want to be involved in supporting young people's interest in music and developing their understanding of music. The Music Institute gives me the opportunity to study a subject that is important to me, and it has developed me both as a musician and as a music teacher. If you choose the Music Institute, you will not be disappointed. The quality of teaching is first class, and the learning environment is second to none. I would recommend the Music Institute to anyone interested in music!  - Jutta, 26 | I myself have been in one band or another and played everything from polka to the heaviest heavy metal. Music has always been a momentary escape from reality for me, because somehow in the flow of playing, all your worries and troubles just disappear. The Music Institute has exposed me to styles of music that were completely unknown to me and has given me a lot of experience that I could probably never have had anywhere else. My time at the Music Institute has been the best of my life, and I hope it never ends. I am absolutely sure that if you apply here, you will definitely not be disappointed.  - Stefan, 38 | Well, it's been a few years since I was at the Music Institute, but I still remember my time there fondly. I taught both music theory and violin there for almost 20 years, and before that I was a student there myself. The curriculum and staff have probably changed several times by now, but at least from what I hear from acquaintances, it is still as good as it was when I was there. At this age, however, it is the friends I made there that I remember most. We still keep in touch with a few of them, even though we all live in different parts of Finland.  - Ritva, 77 |
| --- | --- | --- |
| "I would recommend the Music Institute to anyone" | "My time at the Music Institute has been the best of my life" | "I still remember my time there fondly" |

| **S7 Table** | | | | | | | |
| --- | --- | --- | --- | --- | --- | --- | --- |
| *Factor Loadings, Variances and Error Correlations – COVID-19 (Pretest Measures)* | | | | | | | |
|  |  | Unstandardized | | | Standardized | | |
| Factor | Item | Estimate | SE | Estimate | | SE | *R*^2^ |
| Factor loadings | | | | | | | |
| CVaccAtt (all items) | Vaccination effort | 1.000 | - | 0.090 | | 0.038 | 0.008 |
|  | Vaccine safety | -16.971 | 7.176 | -0.927 | | 0.012 | 0.859 |
|  | Vaccine efficacy | -16.150 | 6.842 | -0.915 | | 0.016 | 0.836 |
|  | Disease threat | -9.228 | 3.888 | -0.547 | | 0.038 | 0.299 |
|  | Vaccination altruism | -19.584 | 8.251 | -0.939 | | 0.012 | 0.882 |
| CVaccAtt (4 items) | Vaccine safety | 1.000 | - | 0.929 | | 0.012 | 0.863 |
|  | Vaccine efficacy | 0.952 | 0.020 | 0.917 | | 0.016 | 0.841 |
|  | Disease threat | 0.542 | 0.041 | 0.546 | | 0.038 | 0.298 |
|  | Vaccination altruism | 1.147 | 0.029 | 0.935 | | 0.013 | 0.875 |
| Format preference | GStatMost* | 1.000 | - | 0.423 | | 0.032 | 0.179 |
|  | GExpFH | 2.029 | 0.152 | 0.858 | | 0.012 | 0.735 |
|  | GExpEasy | 2.090 | 0.155 | 0.883 | | 0.012 | 0.781 |
|  | GExpInf | 1.671 | 0.130 | 0.706 | | 0.020 | 0.499 |
|  | GStatCont | 2.039 | 0.149 | 0.862 | | 0.012 | 0.743 |
|  | GStatEasy* | 1.666 | 0.113 | 0.704 | | 0.020 | 0.496 |
| Error correlations | | | | | | | |
|  | GStatMost* ~ GStatEasy* | 0.321 | 0.024 | 0.498 | | 0.031 | - |
| Factor variances | | | | | | | |
| CVaccAtt (all items) |  | 3.034 | 2.525 | 1.000 | | - | - |
| CVaccAtt (4 items) |  | 877.922 | 51.37 | 1.000 | | - | - |
| Format preference |  | 0.179 | 0.027 | 1.000 | | - | - |
| *Note.* *Reverse coded item. Vaccination effort = perceived effort of getting a COVID-19 vaccine (change score), Vaccine safety = perceived safety of the COVID-19 vaccine (change score), Vaccine efficacy = perceived efficacy of the COVID-19 vaccine, Disease threat = perceived threat of COVID-19 (change score), Vaccination altruism = perceived importance of getting a COVID-19 vaccine to protect others (change score). | | | | | | | |

| **S7 Fig** |
| --- |
| *Response Distributions for Vaccine Attitude Items (Pre-Intervention) – COVID-19* |
| 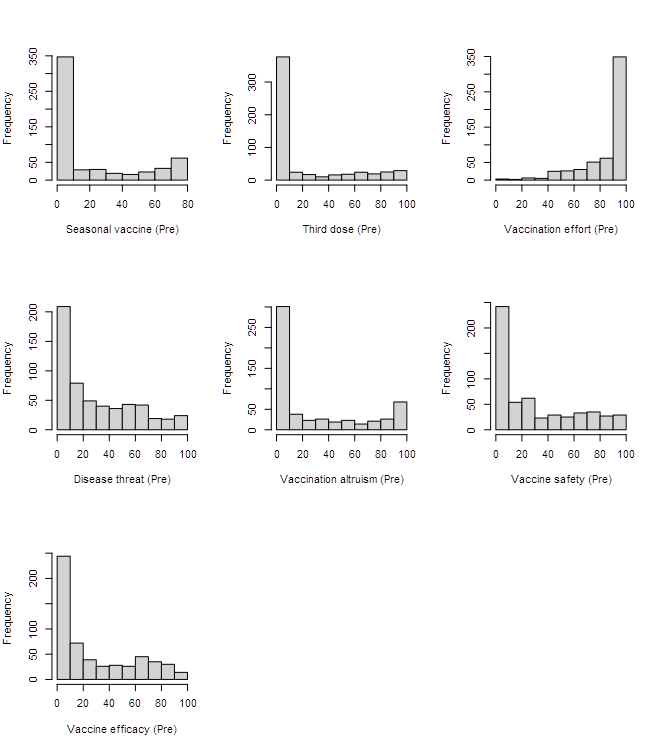 |
| *Note.* Pre = pre intervention measure. Seasonal vaccine = intention to take a seasonal COVID-19, Third dose = intention to take a third COVID-19 vaccine dose, Vaccination effort = perceived effort of getting a COVID-19 vaccine, Vaccine safety = perceived safety of COVID-19 vaccines, Vaccine efficacy = perceived efficacy of COVID-19 vaccines, Disease threat = perceived threat of COVID-19, Vaccination altruism = perceived importance of getting a COVID-19 vaccine to protect others. |

| **S8 Fig** |
| --- |
| *Response Distributions for Format Preference Items – COVID-19* |
| 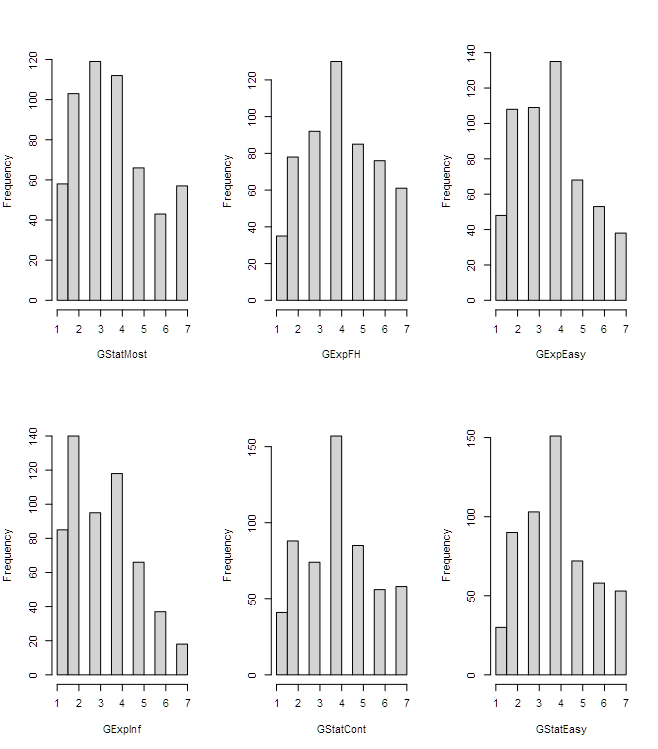 |
| *Note.* GStatMost = “I mostly make decisions about my health based on the statistical information available.”; GExpFH = “I think people's first-hand experiences tell me more about the safety of a medical procedure than statistical research results.”; GExpEasy = “It is easier for me to make decisions about my health based on other people's experiences than on statistical information.”; GExpInf = “Individual people's experiences have a big influence on my health-related decisions.”; GStatCont = “When faced with statistical data that contradicts people's experiences, I prefer to trust people's reported experiences.”; GStatEasy = “I find it easier to make health decisions based on statistical information than on other people's experiences.”. |

| **S8 Table** | | | | | |
| --- | --- | --- | --- | --- | --- |
| *ANOVA for Group Differences Between Pretest Scores – COVID-19 (Pretest Measures)* | | | | | |
| Variable | Df | Sum Sq | Mean Sq | F-Value | P-Value |
| Seasonal vaccine | 2 | 1687 | 843.26 | 1.079 | .3406 |
| Third dose | 2 | 923 | 461.57 | 0.452 | .6366 |
| Vaccination effort | 2 | 1029 | 514.71 | 1.3899 | 0.25 |
| Vaccine confidence | 2 | 4025 | 2012.40 | 2.1804 | .114 |
| Disease threat | 2 | 229 | 114.65 | 0.1323 | .8761 |
| Vaccination altruism | 2 | 912 | 456.02 | 0.3447 | .7086 |
| *Note.* Seasonal vaccine = intention to take a seasonal COVID-19 vaccine (change score), Third dose = intention to take a third COVID-19 vaccine dose (change score), Vaccination effort = perceived effort of getting a COVID-19 vaccine (change score), Vaccine confidence = confidence in the COVID-19 vaccine (change score), Disease threat = perceived threat of COVID-19 (change score), Vaccination altruism = perceived importance of getting a COVID-19 vaccine to protect others (change score). | | | | | |

| **S9 Table** | | | | | | | |
| --- | --- | --- | --- | --- | --- | --- | --- |
| *Factor Loadings, Variances and Error Correlations – Influenza (Pretest Measures)* | | | | | | | |
|  |  | Unstandardized | | | Standardized | | |
| Factor | Item | Estimate | SE | Estimate | | SE | *R*^2^ |
| Factor loadings | | | | | | | |
| IVaccAtt (all items) | Vaccination effort | 1.000 | - | 0.100 | | 0.045 | 0.010 |
|  | Vaccine safety | -13.995 | 6.326 | -0.856 | | 0.017 | 0.732 |
|  | Vaccine efficacy | -13.550 | 6.128 | -0.882 | | 0.015 | 0.778 |
|  | Disease threat | -5.501 | 2.536 | -0.492 | | 0.038 | 0.242 |
|  | Vaccination altruism | -13.335 | 6.066 | -0.832 | | 0.015 | 0.692 |
| IVaccAtt (4 items) | Vaccine safety | 1.000 | - | 0.860 | | 0.017 | 0.740 |
|  | Vaccine efficacy | 0.964 | 0.034 | 0.883 | | 0.015 | 0.780 |
|  | Disease threat | 0.389 | 0.035 | 0.489 | | 0.038 | 0.239 |
|  | Vaccination altruism | 0.944 | 0.040 | 0.828 | | 0.016 | 0.686 |
| Format preference | GStatMost* | 1.000 | - | 0.494 | | 0.030 | 0.244 |
|  | GExpFH | 1.769 | 0.102 | 0.875 | | 0.011 | 0.765 |
|  | GExpEasy | 1.841 | 0.108 | 0.910 | | 0.009 | 0.828 |
|  | GExpInf | 1.659 | 0.098 | 0.820 | | 0.015 | 0.673 |
|  | GStatCont | 1.665 | 0.097 | 0.823 | | 0.015 | 0.678 |
|  | GStatEasy* | 1.570 | 0.080 | 0.776 | | 0.017 | 0.603 |
| Error correlations | | | | | | | |
|  | GStatMost* ~ GStatEasy* | 0.269 | 0.023 | 0.490 | | 0.033 | - |
| Factor variances | | | | | | | |
| IVaccAtt (all items) |  | 4.132 | 3.734 | 1.000 | | - | - |
| IVaccAtt (4 items) |  | 817.786 | 58.396 | 1.000 | | - | - |
| Format preference |  | 0.244 | 0.029 | 1.000 | | - | - |
| *Note.* *Reverse coded item. Vaccination effort = perceived effort of getting the influenza vaccine, Vaccine safety = perceived safety of the influenza vaccine, Vaccine efficacy = perceived efficacy of the influenza vaccine, Disease threat = perceived threat of influenza, Vaccination altruism = perceived importance of getting the influenza vaccine to protect others, Frustration = frustration caused by the intervention, Message relevance, perceived relevance of the intervention material, and Message helpfulness = perceived helpfulness of the intervention material. | | | | | | | |

| **S9 Fig** |
| --- |
| *Response Distributions for Vaccine Attitude Items (Pre-Intervention) – Influenza* |
| 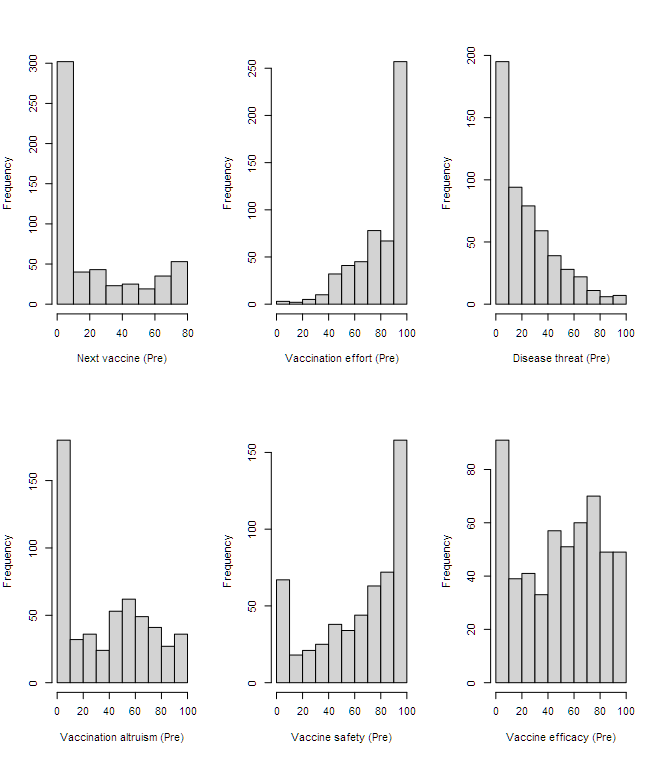 |
| *Note.* Pre = pre intervention measure. Next vaccine = intention to take the next seasonal influenza vaccine, Vaccination effort = perceived effort of getting the influenza vaccine, Vaccine safety = perceived safety of the influenza vaccine, Vaccine efficacy = perceived efficacy of the influenza vaccine, Disease threat = perceived threat of influenza, Vaccination altruism = perceived importance of getting the influenza vaccine to protect others. |

| **S10 Fig** |
| --- |
| *Response Distributions for Format Preference Items – Influenza Experiment* |
| 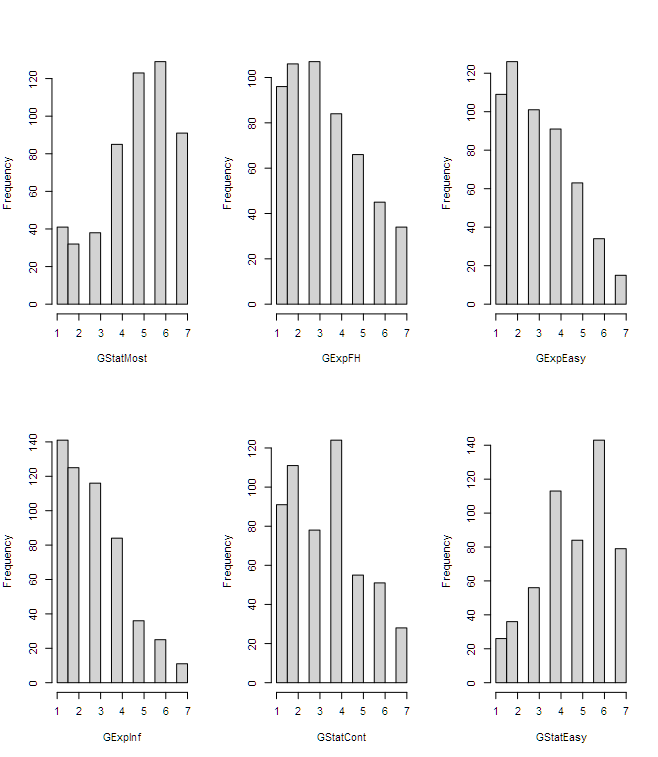 |
| *Note.* GStatMost = “I mostly make decisions about my health based on the statistical information available.”; GExpFH = “I think people's first-hand experiences tell me more about the safety of a medical procedure than statistical research results.”; GExpEasy = “It is easier for me to make decisions about my health based on other people's experiences than on statistical information.”; GExpInf = “Individual people's experiences have a big influence on my health-related decisions.”; GStatCont = “When faced with statistical data that contradicts people's experiences, I prefer to trust people's reported experiences.”; GStatEasy = “I find it easier to make health decisions based on statistical information than on other people's experiences.”. |

| **S10 Table** | | | | | | |
| --- | --- | --- | --- | --- | --- | --- |
| **ANOVA for Group Differences Between Pretest Scores – Influenza (Pretest Measures)** | | | | | | |
| Variable | Df | Sum Sq | Mean Sq | F-Value | P-Value |  |
| Next vaccine | 2 | 1716 | 858.00 | 1.1603 | 0.3142 |  |
| Vaccination effort | 2 | 307 | 153.44 | 0.3699 | 0.691 |  |
| Vaccine confidence | 2 | 1223 | 611.66 | 0.649 | 0.523 |  |
| Disease threat | 2 | 3 | 1.33 | 0.0026 | 0.9974 |  |
| Vaccination altruism | 2 | 491 | 245.28 | 0.2302 | 0.7944 |  |
| *Note.* Next vaccine = intention to take the next seasonal influenza vaccine, Vaccination effort = perceived effort of getting the influenza vaccine, Vaccine safety = perceived safety of the influenza vaccine, Vaccine efficacy = perceived efficacy of the influenza vaccine, Disease threat = perceived threat of influenza, Vaccination altruism = perceived importance of getting the influenza vaccine to protect others. | | | | | |  |

| **S11 Table** | | | | | | | | | | | | |
| --- | --- | --- | --- | --- | --- | --- | --- | --- | --- | --- | --- | --- |
| *Results From the Post Hoc T-Test on Vaccination Intentions Between Self-Reported Intervention Effects* | | | | | | | | | | | | |
| Variable | Statistics | | | | Anecdotes | | | | Control | | | |
|  | *M-Neg* | *M-Pos* | *t* | *p* | *M-Neg* | *M-Pos* | *t* | *p* | *M-Neg* | *M-Pos* | *t* | *p* |
| Seasonal COVID-19 vaccine | 6.79 | 29.04 | -5.40 | < .001 | 4.52 | 24.29 | -7.22 | < .001 | 2.38 | 19.71 | -6.96 | < .001 |
| Third COVID-19 vaccine dose | 5.71 | 28.16 | -4.84 | < .001 | 3.16 | 25.38 | -7.66 | < .001 | 2.19 | 20.08 | -6.21 | < .001 |
| Next seasonal influenza vaccine | 4.00 | 25.93 | -4.66 | < .001 | 2.93 | 23.13 | -7.54 | < .001 | 9.45 | 19.94 | -1.50 | .160 |
| *Note.* M-Neg = Mean value for the group that reported that the interventions had decreased their vaccination intentions. M-Pos = Mean value for the group that reported that the interventions had either increased or not affected their vaccination intentions. | | | | | | | | | | | | |

| **S11 Fig** |
| --- |
| *Response Distributions for Vaccine Attitude Items (Post-Intervention) – COVID-19* |
| 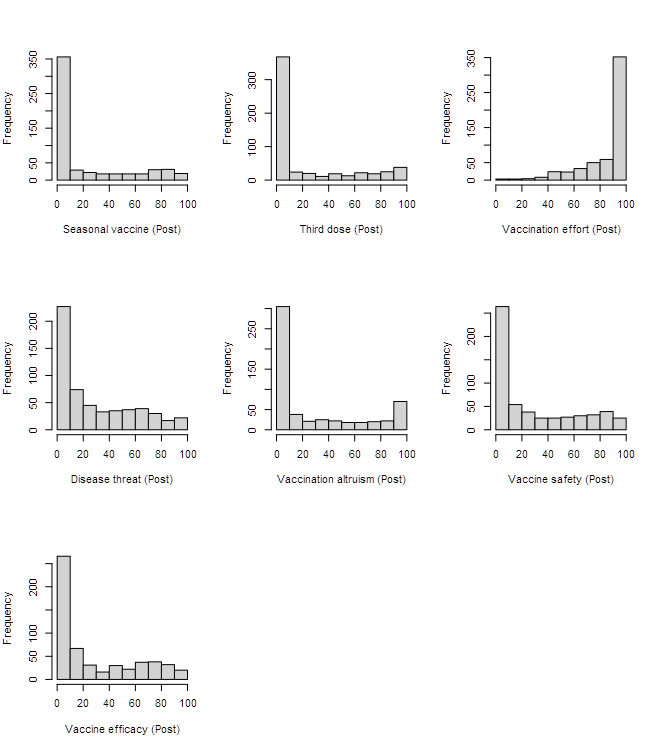 |
| *Note.* Post = post intervention measure. Seasonal vaccine = intention to take a seasonal COVID-19, Third dose = intention to take a third COVID-19 vaccine dose, Vaccination effort = perceived effort of getting a COVID-19 vaccine, Vaccine safety = perceived safety of COVID-19 vaccines, Vaccine efficacy = perceived efficacy of COVID-19 vaccines, Disease threat = perceived threat of COVID-19, Vaccination altruism = perceived importance of getting a COVID-19 vaccine to protect others. |

| **S12 Fig** |
| --- |
| *Response Distributions for Vaccine Attitude Items (Change Score) – COVID-19* |
| 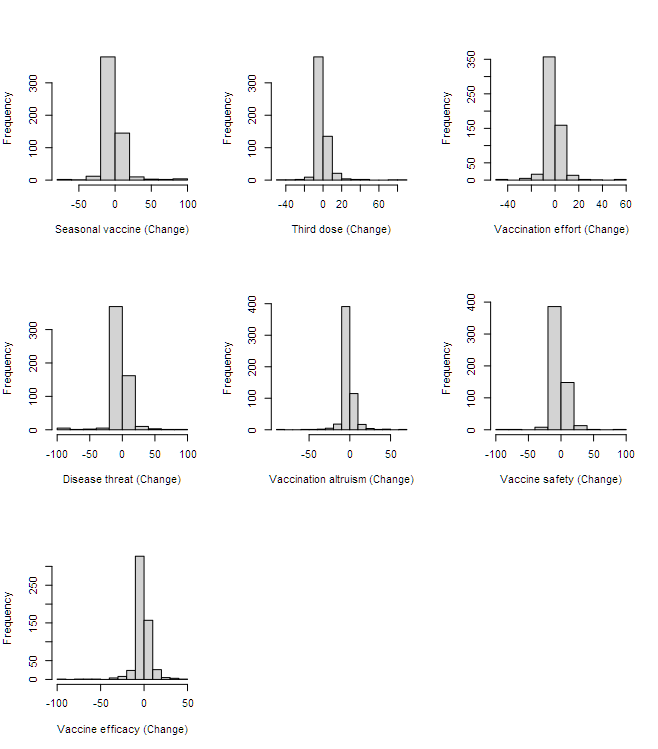 |
| *Note.* Change = change score (post-pre). Seasonal vaccine = intention to take a seasonal COVID-19, Third dose = intention to take a third COVID-19 vaccine dose, Vaccination effort = perceived effort of getting a COVID-19 vaccine, Vaccine safety = perceived safety of COVID-19 vaccines, Vaccine efficacy = perceived efficacy of COVID-19 vaccines, Disease threat = perceived threat of COVID-19, Vaccination altruism = perceived importance of getting a COVID-19 vaccine to protect others. |

| **S13 Fig** |
| --- |
| *Response Distributions for Vaccine Attitude Items (Post-Intervention) – Influenza* |
| 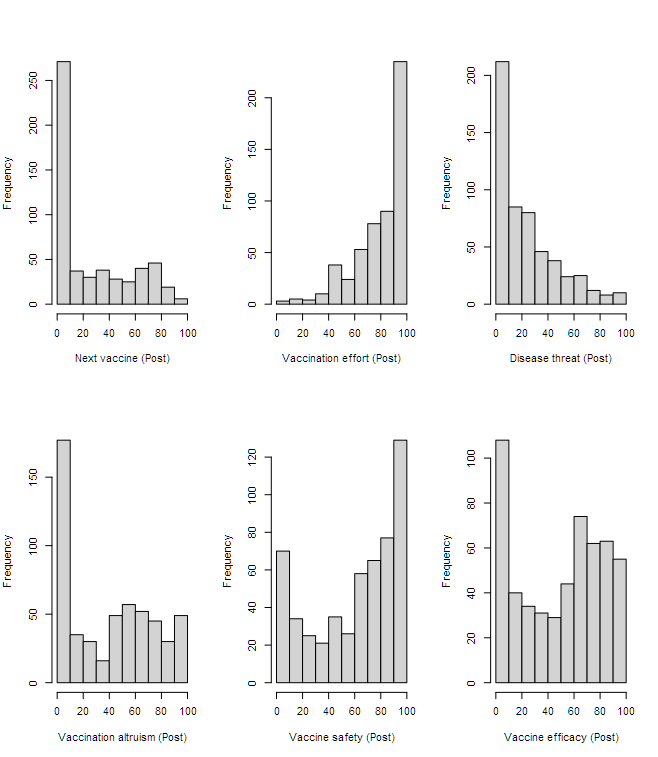 |
| *Note.* Post = post intervention measure. Next vaccine = intention to take the next seasonal influenza vaccine, Vaccination effort = perceived effort of getting the influenza vaccine, Vaccine safety = perceived safety of the influenza vaccine, Vaccine efficacy = perceived efficacy of the influenza vaccine, Disease threat = perceived threat of influenza, Vaccination altruism = perceived importance of getting the influenza vaccine to protect others. |

| **S14 Fig** |
| --- |
| *Response Distributions for Vaccine Attitude Items (Change Score) – Influenza* |
| 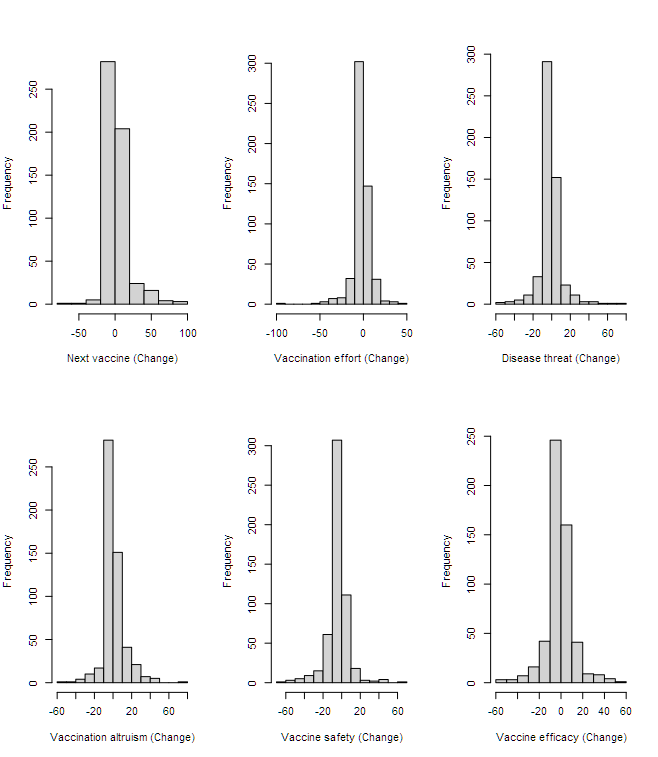 |
| *Note.* Change = change score (post-pre). Next vaccine = intention to take the next seasonal influenza vaccine, Vaccination effort = perceived effort of getting the influenza vaccine, Vaccine safety = perceived safety of the influenza vaccine, Vaccine efficacy = perceived efficacy of the influenza vaccine, Disease threat = perceived threat of influenza, Vaccination altruism = perceived importance of getting the influenza vaccine to protect others. |
