# Supplementary material for: Tailoring interventions to suit self-reported format preference does not decrease vaccine hesitancy
Source: PLoS One. 2023 Mar 21;18(3):e0283030. doi: 10.1371/journal.pone.0283030 (PMC10030039; doi:10.1371/journal.pone.0283030)
Supplement: S3 File — (DOCX) [file pone.0283030.s003.docx]

**S3 File. Supporting information for the format preference scale validation studies.**

Across three studies, we first created a short scale for format preference and validated it by investigating its associations with theoretically related constructs. Before using the scale in interventions with existing diseases and vaccines, we tested whether the scale predicted how susceptible people were to pro- and anti-vaccine anecdotal information related to hypothetical diseases and vaccines. This was done because previous studies have demonstrated a negativity bias concerning information about vaccines (Betsch et al., 2015). Prior to data collection, the Research Ethics Committee in Psychology and Logopedics of Åbo Akademi University gave ethical permission for the three studies.

**Study 1 - Scale Construction**

***Method***

**Participants and Procedure.** In March 2021, a post with a link to an electronic survey was shared and marketed to Facebook and Reddit users in Finland who have reported being 18 years old or older. Altogether, 473 people completed the survey. No compensation was offered for answering the survey. See Table S1 for the sample demographics.

| **S1 Table** |  |  |
| --- | --- | --- |
| Sample Demographics – Studies 1–3 | | |
| **Variable** | ***n*** | **%** |
| Study 1 |  |  |
| Age |  |  |
| 18 – 29 | 110 | 23.26 |
| 30 – 39 | 67 | 14.16 |
| 40 – 49 | 91 | 19.24 |
| 50 – 59 | 97 | 20.51 |
| 60 – 69 | 74 | 15.64 |
| 70 – 79 | 32 | 6.77 |
| 80 – 89 | 2 | 0.42 |
| Missing | 0 | 0 |
| Gender |  |  |
| Male | 111 | 23.42 |
| Female | 346 | 73.00 |
| Other | 7 | 1.48 |
| Did not want to report | 9 | 1.90 |
| Missing | 0 | 0 |
|  |  |  |
| Study 2 |  |  |
| Age |  |  |
| 18 – 29 | 44 | 11.40 |
| 30 – 39 | 69 | 17.88 |
| 40 – 49 | 113 | 29.27 |
| 50 – 59 | 67 | 17.36 |
| 60 – 69 | 42 | 10.88 |
| 70 – 79 | 10 | 2.59 |
| 80 – 89 | 0 | 0 |
| Missing | 41 | 10.62 |
| Gender |  |  |
| Male | 51 | 13.21 |
| Female | 315 | 81.61 |
| Other | 8 | 2.07 |
| Did not want to report | 9 | 2.33 |
| Missing | 3 | 0.78 |
| Education |  |  |
| Lower | 115 | 29.79 |
| Higher | 269 | 69.69 |
| Other | 1 | 0.26 |
| Missing | 1 | 0.26 |
| Region |  |  |
| Uusimaa | 99 | 25.65 |
| Varsinais-Suomi | 109 | 28.24 |
| Other | 178 | 46.11 |
| Missing | 0 | 0 |
| Worked as a healthcare worker |  |  |
| Yes | 135 | 34.97 |
| No | 251 | 65.03 |
| Missing | 0 | 0 |
|  |  |  |
| Study 3 |  |  |
| Age |  |  |
| 18 – 29 | 70 | 24.65 |
| 30 – 39 | 54 | 19.01 |
| 40 – 49 | 30 | 10.56 |
| 50 – 59 | 16 | 5.63 |
| 60 – 69 | 3 | 1.06 |
| 70 – 79 | 0 | 0 |
| 80 – 89 | 0 | 0 |
| Missing | 111 | 39.08 |
| Gender |  |  |
| Male | 115 | 40.49 |
| Female | 163 | 57.39 |
| Other | 4 | 1.41 |
| Did not want to report | 1 | 0.35 |
| Missing | 1 | 0.35 |
| Education |  |  |
| Lower | 84 | 29.58 |
| Higher | 199 | 70.07 |
| Other | 0 | 0 |
| Missing | 1 | 0.35 |
| Region |  |  |
| Uusimaa | 97 | 34.15 |
| Varsinais-Suomi | 59 | 20.77 |
| Pirkanmaa | 31 | 10.92 |
| Other | 95 | 33.45 |
| Missing | 1 | 0.35 |
| Worked as a healthcare worker |  |  |
| Yes | 79 | 27.82 |
| No | 202 | 71.13 |
| Missing | 2 | 0.70 |
|  |  |  |

**Measures.** Table S2 shows a full list of the constructed format preference -items. Table S3 and Figure S1 show format preference item distributions.

| **S2 Table** | |
| --- | --- |
| *All Format preference -Items with Abbreviations* | |
| **Item** | **Abbreviation** |
| I mostly make decisions about my health based on the statistical information available.* | GStatMost |
| I think people's first-hand experiences tell me more about the safety of a medical procedure than statistical research results. | GExpFH |
| It is easier for me to make decisions about my health based on other people's experiences than on statistical information. | GExpEasy |
| Individual people's experiences have a big influence on my health-related decisions. | GExpInf |
| When faced with statistical data that contradicts people's experiences, I prefer to trust people's reported experiences. | GStatCont |
| I find it easier to make health decisions based on statistical information than on other people's experiences.* | GStatEasy |
| Statistical data on the safety and efficacy of vaccines is not enough for me to make an informed decision on whether to take a vaccine. | VStatNo |
| When making vaccination decisions, I tend to rely more on statistical information than on the personal experiences of others.* | VStatRely |
| I prefer to base my vaccination decisions on people's self-reported experiences, rather than on statistical research results. | VExpPrefSelf |
| Another person's negative experience with a vaccine can make me hesitate to take that vaccine. | VExpHes |
| I prefer to base my vaccination decisions on probabilities and statistical information, rather than on my own and others' personal experiences with vaccines.* | VStatPref |
| I prefer to base my vaccination decisions on what other people think about the safety and effectiveness of the vaccine, rather than on statistical data on these issues. | VExpPrefAtt |
| *Note.* Response scale: 1 = strongly disagree to 7 = strongly agree.  *Reversed item | |

| **S3 Table** | | | | |
| --- | --- | --- | --- | --- |
| *Format preference Item Distributions – Study 1* | | | | |
| Statement | Mean | SD | Skewness | Kurtosis |
| GStatMost* | 3.02 | 1.55 | 0.80 | 0.09 |
| GExpFH | 2.43 | 1.40 | 1.02 | 0.37 |
| GExpEasy | 2.69 | 1.40 | 0.66 | -0.33 |
| GExpInf | 2.55 | 1.39 | 0.88 | 0.14 |
| GStatCont | 2.51 | 1.45 | 0.98 | 0.42 |
| GStatEasy* | 2.84 | 1.53 | 0.79 | 0.06 |
| VStatNo | 2.34 | 1.74 | 1.40 | 0.87 |
| VStatRely* | 2.04 | 1.43 | 1.85 | 3.11 |
| VExpPrefSelf | 1.78 | 1.17 | 2.17 | 5.26 |
| VExpHes | 2.13 | 1.54 | 1.50 | 1.54 |
| VStatPref* | 2.21 | 1.48 | 1.61 | 2.26 |
| VExpPrefAtt | 1.82 | 1.24 | 2.21 | 5.33 |
| *Note.* *Reverse coded item. | | | | |

| **S1 Figure** |
| --- |
| *Violin Plots of Mean Scores and Distributions of the Format Preference Items – Study 1* |
| 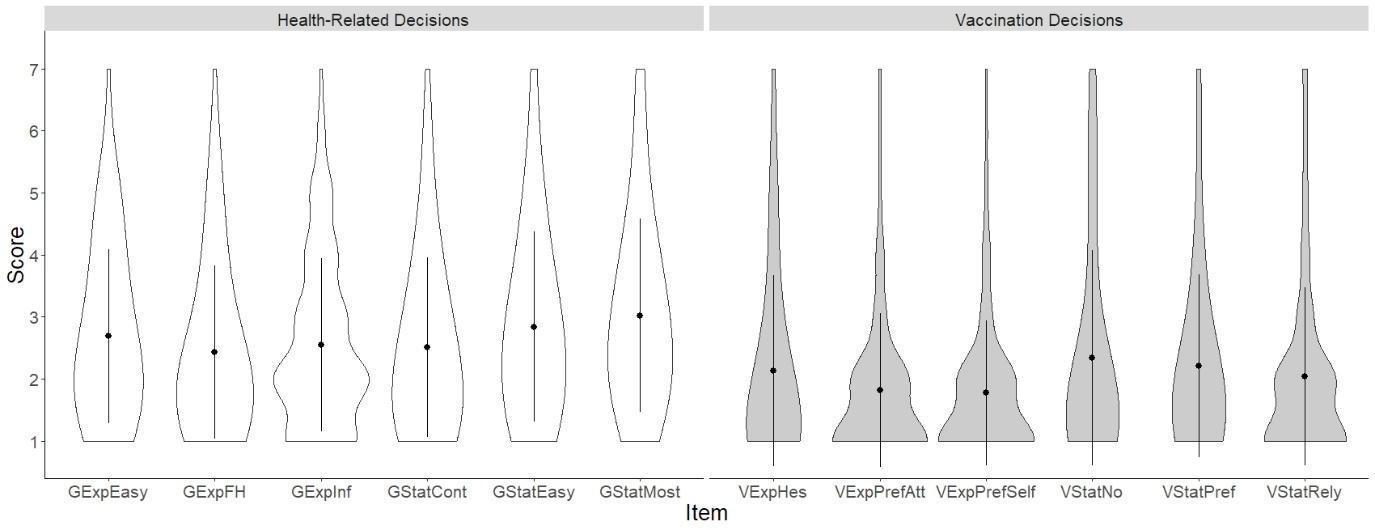 |
| *Note.* The figure shows the means (circles), standard deviations (bars), and the frequency distributions (outer edges) of the responses to the statements about the reliance on statistics versus anecdotes. The figure shows that most people tend to rely on statistical information (lower scores) while making health or vaccination decisions, rather than relying on anecdotal information (higher scores). |

***Results***

In short, due to poor fit, the vaccination-specific items were excluded from the model. The final CFA model included the first six indicators (GStatMost, GExpFH, GExpEasy, GExpInf, GStatCont, and GStatEasy) specified to load on one latent variable (format preference). The model also included an error correlation between the two reverse-coded items (GStatMost and GStatEasy) and the items GExpFH and GStatCont. Due to the response variables being ordinal, robust WLS (WLSMV) estimation was employed (Brown, 2015). Cronbach’s alpha was used to estimate internal consistency. All fit indices showed good fit for the final six-item-model (CFI = 0.995, TLI = 0.989, SRMR = .018, RMSEA = .070). All standardized factor loadings were equal to or larger than .50 (Table S4). Cronbach’s alpha for the six items was also good (Cronbach’s α = .85).

| **S4 Table** | | | | | |
| --- | --- | --- | --- | --- | --- |
| *Factor Loadings, Variances and Error Correlations – Study 1* | | | | | |
|  | Unstandardized | | Standardized | | |
| Item | Estimate | SE | Estimate | SE | *R*^2^ |
| Factor loadings | | | | | |
| GStatMost* | 1.00 | - | .50 | .04 | .25 |
| GExpFH | 1.52 | .11 | .76 | .02 | .58 |
| GExpEasy | 1.72 | .12 | .86 | .02 | .75 |
| GExpInf | 1.53 | .11 | .76 | .02 | .58 |
| GStatCont | 1.40 | .10 | .70 | .03 | .49 |
| GStatEasy* | 1.46 | .09 | .72 | .03 | .52 |
| Error correlations | | | | | |
| GStatMost* ~ GStatEasy* | .27 | .03 | .44 | .04 | .19 |
| GExpFH ~ GStatCont | .15 | .02 | .33 | .04 | .11 |
| Factor variances | | | | | |
| Latent variable |  |  |  |  |  |
| Format preference | .25 | .04 | 1.00 | - | - |
|  |  |  |  |  |  |
| *Note.* *Reverse coded item. | | | | | |

**Studies 2 and Study 3 – Scale Validation**

To validate the 6-item format preference scale, we conducted two data collections. In both samples, we retested the scale’s reliability and factor structure, assessed the validity of the scale by testing how it related to constructs that are theoretically expected to be associated with format preference, and investigated how accurately the format preference scale predicted participant susceptibility to anecdotal vaccine-related information. We explored susceptibility to pro- and anti-vaccine anecdotes separately, in order to get more detailed information on the relationship between the format preference scale and the susceptibility to anecdotal information. We expected that individuals with a stronger preference for anecdotes would be more positively affected by the pro-vaccine anecdotes and more negatively affected by the anti-vaccine anecdotes respectively.

***Method***

**Participants.** A post with a link to an electronic survey was shared and marketed on Facebook in May 2021, and a second time in July 2021. The surveys were marketed to Facebook users in Finland who have reported being 18 years old or older. Altogether 403 people participated in the first data collection and 298 in the second. Respondents were not offered any compensation for completing the survey. After omitting 17 participants in the first sample and 14 in the other due to failed attention checks, the final sample sizes were 386 and 284 respectively. See Table S1 for the sample demographics.

**Procedure.** In both data collections the participants first gave their informed consent and provided demographic information. Then, they proceeded to an experimental task measuring susceptibility to anecdotal information. After that, they filled out forms related to trust in vaccines, trust in health authorities, conspiracy mentality, need for affect, and numeracy. Finally, they filled out the format preference scale. The order of the questionnaire was the same for all participants (see Figure S2 and S3 for an overview of the data collection procedures).

| **S2 Figure**  Overview of Procedure – Study 2 | | | | |
| --- | --- | --- | --- | --- |
|  | | | | |
| **Group P** |  | **Group N** |  | **Group C** |
|  | | | | |
| Demographic information | | | | |
|  | | | | |
| Statistical information  *Disease 1* | | | | |
|  | | | | |
| Pre-measures  *Disease 1* | | | | |
|  |  |  |  |  |
| Positive anecdotal information  *Disease 1* |  | Negative anecdotal information  *Disease 1* |  | Control information  *Disease 1* |
|  | | | | |
| Post-measures  *Disease 1* | | | | |
|  | | | | |
| Statistical information  *Disease 2* | | | | |
|  | | | | |
| Pre-measures  *Disease 2* | | | | |
|  |  |  |  |  |
| Positive anecdotal information  *Disease 2* |  | Negative anecdotal information  *Disease 2* |  | Control information  *Disease 2* |
|  | | | | |
| Post-measures  *Disease 2* | | | | |
|  | | | | |
| Statistical information  *Disease 3* | | | | |
|  | | | | |
| Pre-measures  *Disease 3* | | | | |
|  |  |  |  |  |
| Positive anecdotal information  *Disease 3* |  | Negative anecdotal information  *Disease 3* |  | Control information  *Disease 3* |
|  | | | | |
| Post-measures  *Disease 3* | | | | |
|  | | | | |
| Trust in Health Authorities  Vaccine Confidence  Need for Affect  Numeracy  Format preference | | | | |
|  | | | | |
| *Note.* Steps color coded as grey were shared across all groups. | | | | |

| **S3 Figure**  *Overview of Procedure – Study 3* | | | | |
| --- | --- | --- | --- | --- |
|  | | | | |
| **Condition 1** |  | **Condition 2** |  | **Condition 3** |
|  | | | | |
| Demographic information | | | | |
|  | | | | |
| Statistical information  *Disease 1* |  | Statistical information  *Disease 2* |  | Statistical information  *Disease 3* |
|  | | | | |
| Pre-measures | | | | |
|  |  |  |  |  |
| Positive anecdotal information  *Disease 1* |  | Control information  *Disease 2* |  | Negative anecdotal information  *Disease 3* |
|  | | | | |
| Post-measures | | | | |
|  | | | | |
| Statistical information  *Disease 2* |  | Statistical information  *Disease 3* |  | Statistical information  *Disease 1* |
|  | | | | |
| Pre-measures | | | | |
|  |  |  |  |  |
| Negative anecdotal information  *Disease 2* |  | Positive anecdotal information  *Disease 3* |  | Control information  *Disease 1* |
|  | | | | |
| Post-measures | | | | |
|  | | | | |
| Statistical information  *Disease 3* |  | Statistical information  *Disease 1* |  | Statistical information  *Disease 2* |
|  | | | | |
| Pre-measures | | | | |
|  |  |  |  |  |
| Control information  *Disease 3* |  | Negative anecdotal information  *Disease 1* |  | Positive anecdotal information  *Disease 2* |
|  | | | | |
| Post-measures | | | | |
|  | | | | |
| Trust in Health Authorities  Vaccine Confidence  Need for Affect  Numeracy  Format preference | | | | |
|  | | | | |
| *Note.* Steps color coded as grey were shared across all conditions. | | | | |

**Materials.**

***Susceptibility to Anecdotal information.*** Susceptibility to anecdotal information was measured with a modified version of a task used by (Betsch et al., 2011). In this task, the participants were first asked to read statistical information on the threat of a hypothetical disease and the safety and efficacy of a hypothetical vaccine. This information was presented as short texts in an image of a made-up health authority webpage (called “The Institute of Finnish Health”), designed to closely resemble a real health authority webpage (see Figure S4). All texts on the made-up health authority webpage were in favor of the vaccine. The severity of the disease and the safety and efficacy of the vaccine were calibrated to avoid a ceiling effect where all individuals would be willing to take the vaccine (e.g., the disease was not presented as extremely threatening and the vaccine not as 100% effective).

| **S4 Figure** | |
| --- | --- |
| *Example Images of the Made-Up Webpages Used in Preparation Studies 2 and 3.* | |
| Health authority webpage | Discussion forum |
| 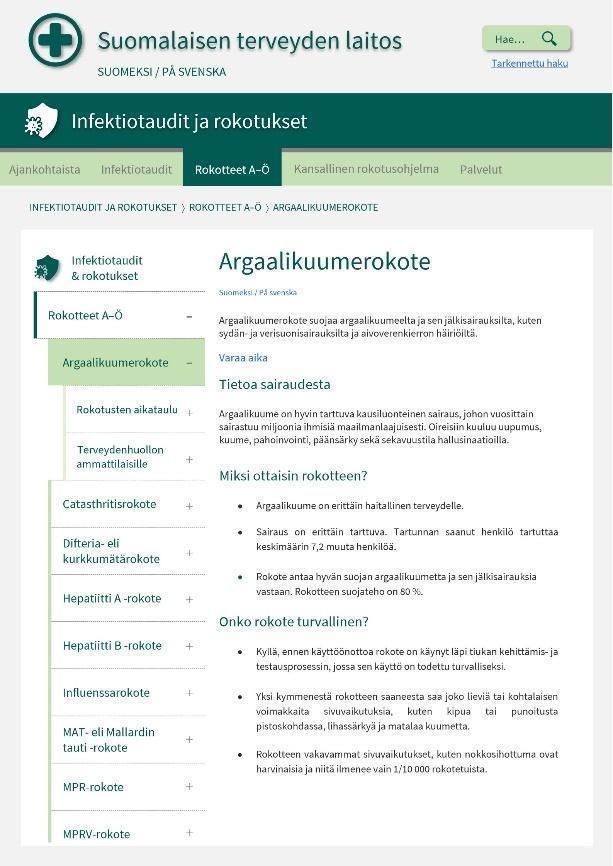 | 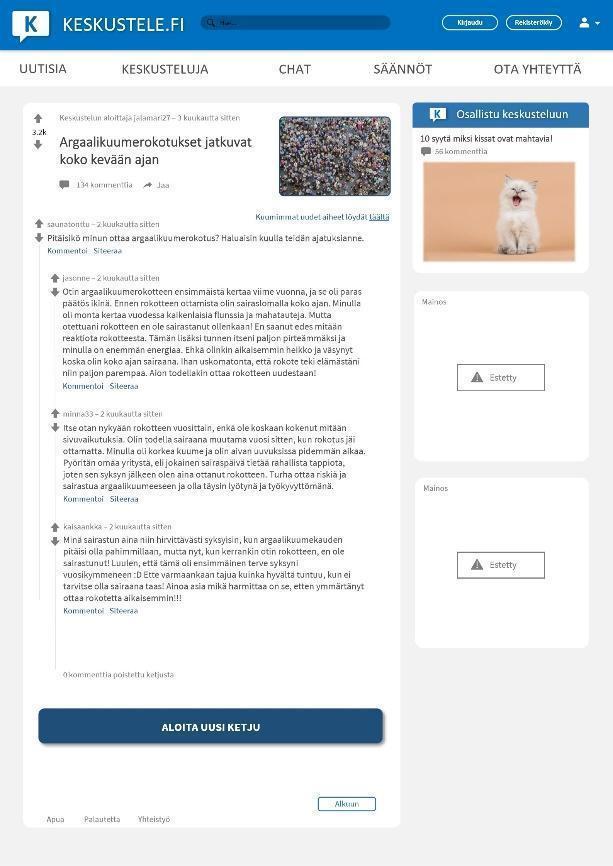 |
|  |  |

Participants were then presented with the pre-anecdote measures (see details below), which measured their perceptions about the hypothetical disease and vaccine. These served as baseline measures against which the post-measures were compared.

Next, the participants were presented with an image of a made-up discussion forum (called “Keskustele.fi”; English translation: “Discuss.fi”) containing personal anecdotes about the hypothetical disease and its vaccine (see Figure S4). The Pro-vaccine anecdotes involved strong positive feelings toward the vaccine, for example people praising the effectiveness of the vaccine or reporting that they did not get any side effects from it. The Anti-vaccine anecdotes were personal stories with strong negative feelings toward the vaccine, such as anecdotes about vaccinated people who had contracted the disease or had suffered severe side effects from the vaccine. The Control anecdotes were unrelated to vaccines, such as people sharing cat and dog breed experiences. Data collection 1 followed a between-subject design, where the participants were randomly assigned to the Pro-vaccine group, the Anti-vaccine group, and the Control group. Data collection 2, on the other hand, followed a within-subjects design, meaning that all participants received all three anecdote types. Using a Graeco-Latin square design, three unique sequences were devised so that both the order of the diseases and the disease-anecdote type pairs were distinct for the three sequences.

After each webpage, participants were asked a multiple-choice question to check their attention, resulting in a total of six attention-checks. The questions related to the health authority webpages probed the name of the disease, whereas the questions related to the discussion forums targeted the content of the discussions. An incorrect answer on any of the attention check questions, resulted in that person’s exclusion from all analyses.

Lastly, respondents were presented with the post-anecdote measures to assess to which degree the respondents were affected by the anecdotal information. A greater change in either direction between pre- and post-anecdote measures was interpreted as a stronger susceptibility to anecdotal information. The task was done three times in a row, with three different made-up diseases and vaccines.

***Pre- and Post-Anecdote Measures.*** All pre- and post-anecdote measures were assessed on a slider ranging from 0 to 100. The numeric anchors were not visible to the participants. The pointer was set to start from the midpoint of the slider (= 50). *Vaccination intention* was measured with the question “Given all the information presented so far, how likely would you be to take the vaccine against [disease]?” (0 = *very unlikely;* 100 = *very likely*); *Perceived disease threat* was measured with the question “Considering all the information presented so far, how big a threat do you consider [disease] to be to your health?” (0 = *not a threat at all;* 100 = *very threatening*); *Perceived vaccine safety* was assessed with the question “Considering all the information presented so far, how safe do you think the [vaccine] is?” (0 = *not safe at all;* 100 = *very safe*); and *Perceived vaccine efficacy*, was measured with the question “Considering all the information presented so far, how effective do you find the [vaccine] to be?” (0 = *not at all effective*; 100 = *very effective*).

***Trust in Vaccines.*** Trust in vaccines was measured with the statement “I am completely confident that vaccines are safe.” (Betsch et al., 2018) that was answered on a Likert scale ranging from 1 = *strongly disagree* to 7 = *strongly agree*.

***Trust in Health Authorities.*** Trust in health authorities was measured with the following four statements: 1) “I trust the information I receive from health authorities about vaccines.”, 2) “Health authorities would not recommend vaccines that are not safe to take.”, 3) “I prefer other sources of information to official health authorities.”, and 4) “Health authorities have the public's best interests at heart.”. The former two statements were originally created by (Karlsson et al., 2019) and the latter two were created for the present study. Participants responded to these items on a Likert scale ranging from 1 = *strongly disagree* to 7 = *strongly agree*.

***Conspiracy Mentality.*** Conspiracy mentality was measured with the five-item Conspiracy Mentality Questionnaire (Bruder et al., 2013). The questionnaire consisted of statements, such as: “I think that many very important things happen in the world, which the public is never informed about.”, that were answered on an eleven-point fully labeled scale from 0% = *certainly not* to 100% = *certain*.

***Need for Affect.*** Need for affect was measured with the short form of the Need for Affect Questionnaire (Appel et al., 2012). The questionnaire consisted of ten items (e.g., “If I reflect on my past, I see that I tend to be afraid of feeling emotions”) which were answered on a Likert scale ranging from -3 = *strongly disagree* to 3 = *strongly agree*. The questionnaire measured two factors – emotion approach (a tendency to approach emotion inducing situations) and emotion avoidance (tendency to avoid emotion inducing situations).

***Numeracy.*** Numeracy was measured with the one-item version of the Berlin Numeracy test (see Cokely et al., 2012). Participants were asked to answer the question: “Out of 1,000 people in a small town 500 are members of a choir. Out of these 500 members in the choir 100 are men. Out of the 500 inhabitants that are not in the choir 300 are men. What is the probability that a randomly drawn man is a member of the choir?”. Participants were instructed to answer the probability in percent and without using a calculator.

***Format preference.*** Format preference was measured with the final six-item version of the scale (see Study 1).

**Statistical Analyses.**

***Measurement Models and Internal Consistency.*** In both samples, we conducted separate confirmatory factor analyses for the format preference scale (six indicators; error correlation between reverse-scored items), trust in health authorities (four indicators), conspiracy mentality (five indicators; error correlation between the first two items), emotion approach (five indicators), and emotion avoidance (five indicators) measures, to confirm that their respective indicators formed factors and that we could use the mean scores for these measures in later analyses. We also assessed the internal consistency of the items with Cronbach’s alpha.

***Associations between Format preference and Theoretically Associated Constructs.*** To validate the format preference scale against constructs that can be expected to be closely related to format preference, we conducted bivariate correlation analyses between the format preference scale and trust in vaccines, trust in health authorities, conspiracy mentality, emotion approach, and emotion avoidance in both samples. The format preference score was calculated as the mean of the six format preference items, and the score was standardized before analysis. The scores for trust in health authorities, conspiracy mentality, and need for affect subfactors were calculated as the mean of their respective indicators. All reversed items were reverse coded before the analyses. We analyzed the association between the format preference scale and numeracy using the Wilcoxon rank-sum test. Numeracy was coded dichotomously so that an incorrect answer resulted in the value 0 (low numeracy) whereas a correct answer resulted in the value 1 (high numeracy).

***Does Format Preference Predict Susceptibility to Anecdotal Information?*** Finally, we examined whether format preference scores were associated with the susceptibility to anecdotal information. The susceptibility to anecdotal information was operationalized as the change in vaccination intention, perceived disease threat, perceived vaccine safety, and perceived vaccine efficacy (the difference between pre- and post-anecdote measures) as a result of having read pro- or anti-vaccine anecdotes. Since the responses to the pre- and post-anecdote measures were not normally distributed in either sample, we conducted the analyses with mean change scores instead of using a repeated-measures analysis. We calculated separate change scores for all four measures, by subtracting the pre-anecdote measure scores from the post-anecdote measure scores. Then we calculated the mean change across the three hypothetical diseases. This resulted in change scores for which larger positive and negative values indicated a greater susceptibility to anecdotal information, whereas a value of zero indicated no susceptibility. For the first sample, we used multiple regression to test if format preference predicts anecdotal bias. We investigated if group moderated the effect of format preference on anecdotal bias, by adding an interaction term between format preference and the anecdote groups as dummy coded predictors with the control group as the reference, with the comparisons being between the Control group and the Pro-vaccine group, and between the Control group and the Anti-vaccine group. The change scores for vaccination intention, perceived disease threat, perceived vaccine safety and perceived vaccine efficacy were analyzed separately as outcome variables, with the anecdote group as a dummy coded predictor (with the Control group as reference), and the format preference scale as a continuous predictor (centered), including the interaction term between anecdote group and format preference in all models. Additionally, we estimated the separate format preference slopes for each statistically significant interaction between format preference and anecdote group.

For the second sample, we analyzed the association between the format preference scale and the effect of anecdote type (pro-vaccine, anti-vaccine, and control) on participants’ perceptions of disease threat, vaccine safety and efficacy, and their intentions to take the vaccines, using linear mixed effect models. The change scores for vaccination intention, perceived disease threat, perceived vaccine safety, and perceived vaccine efficacy were included as outcome variables, the anecdote type as a dummy coded fixed factor (with the control anecdote type as reference), the format preference as a continuous fixed factor (centered), with the interaction term between anecdote type and format preference. Participant was set as a random factor. We also estimated the separate format preference slopes for each anecdote type.

***Results***

**Measurement Models and Internal Consistency.** See Tables S5 and S6 for the full CFA models’ fit indices and Tables S7 and S8 for all factor loadings for Study 2 and Study 3 respectively. In the first sample, the fit indices for the format preference scale, trust in health authorities, and conspiracy mentality models showed excellent fit, with all standardized factor loadings being above .50. All fit indices, except the RMSEA, showed good fit for the emotion approach and emotion avoidance models, with all standardized factor loadings being above .60. Internal consistency was good for all measures: format preference (Cronbach’s α = .87), trust in health authorities (Cronbach’s α = .90), conspiracy mentality (Cronbach’s α = .83), emotion approach (Cronbach’s α = .76), and emotion avoidance (Cronbach’s α = .84).

| **S5 Table** | | | | | | |
| --- | --- | --- | --- | --- | --- | --- |
| *Goodness-of-Fit Indicators for the CFA Models – Study 2* | | | | | | |
| Model | χ^2^ | df | CFI | TLI | SRMR | RMSEA |
| Format preference | 11.108 | 8 | 0.999 | 0.999 | 0.013 | 0.025 |
| Trust in health authorities | 0.142 | 2 | 1.000 | 1.001 | 0.001 | 0.000 |
| Conspiracy mentality | 5.021 | 4 | 1.000 | 0.999 | 0.013 | 0.026 |
| Emotion approach | 21.197 | 5 | 0.988 | 0.975 | 0.039 | 0.092 |
| Emotion avoidance | 41.179 | 5 | 0.985 | 0.969 | 0.037 | 0.137 |
| *Note.* CFI > .90 = good fit, TLI > .90 = good fit, SRMR < .08 = good fit, RMSEA < .08 = good fit. | | | | | | |

| **S6 Table** | | | | | | |
| --- | --- | --- | --- | --- | --- | --- |
| *Goodness-of-Fit Indicators for the CFA Models – Study 3* | | | | | | |
| Model | Χ^2^ | df | CFI | TLI | SRMR | RMSEA |
| Format preference | 21.23 | 8 | 0.997 | 0.995 | 0.022 | 0.077 |
| Trust in health authorities | 2.38 | 2 | 1.000 | 1,000 | 0.007 | 0.026 |
| Conspiracy mentality | 15.80 | 4 | 0.993 | 0.982 | 0.026 | 0.102 |
| Emotion approach | 5.42 | 5 | 1.000 | 0.999 | 0.022 | 0.017 |
| Emotion avoidance | 15.83 | 5 | 0.993 | 0.986 | 0.026 | 0.088 |
| *Note.* CFI > .90 = good fit, TLI > .90 = good fit, SRMR < .08 = good fit, RMSEA < .08 = good fit. | | | | | | |

| **S7 Table** | | | | | | |
| --- | --- | --- | --- | --- | --- | --- |
| *Factor Loadings, Variances and Error Correlations for the CFA Models – Study 2* | | | | | | |
|  |  | Unstandardized | | Standardized | | |
| Latent Variable | Item | Estimate | SE | Estimate | SE | *R*^2^ |
| Factor loadings | | | | | | |
| Format preference | GStatMost* | 1.00 | - | .54 | .04 | .29 |
|  | GExpFH | 1.56 | 0.11 | .84 | .02 | .71 |
|  | GExpEasy | 1.58 | 0.11 | .85 | .02 | .73 |
|  | GExpInf | 1.31 | 0.10 | .71 | .03 | .50 |
|  | GStatCont | 1.45 | 0.10 | .78 | .02 | .61 |
|  | GStatEasy* | 1.53 | 0.09 | .83 | .02 | .69 |
| Trust in health authorities | TrustInfo | 1.00 | - | .93 | .01 | .87 |
|  | TrustRecommend | 0.96 | 0.02 | .89 | .01 | .80 |
|  | TrustOther* | 0.82 | 0.03 | .77 | .03 | .59 |
|  | TrustIntent | 0.95 | 0.02 | .89 | .02 | .79 |
| Conspiracy mentality | CMQ1 | 1.00 | - | .61 | .03 | .37 |
|  | CMQ2 | 1.10 | 0.05 | .67 | .03 | .45 |
|  | CMQ3 | 0.97 | 0.07 | .59 | .03 | .34 |
|  | CMQ4 | 1.48 | 0.08 | .90 | .02 | .80 |
|  | CMQ5 | 1.46 | 0.08 | .88 | .02 | .78 |
| Emotion approach | NFA2 | 1.00 | - | .45 | .04 | .20 |
|  | NFA3 | 1.23 | 0.14 | .55 | .04 | .30 |
|  | NFA5 | 1.83 | 0.18 | .82 | .03 | .67 |
|  | NFA8 | 1.90 | 0.19 | .85 | .03 | .72 |
|  | NFA9 | 1.48 | 0.15 | .66 | .03 | .43 |
| Emotion avoidance | NFA1 | 1.00 | - | .75 | .03 | .56 |
|  | NFA4 | 1.16 | 0.04 | .86 | .02 | .74 |
|  | NFA6 | 0.98 | 0.05 | .73 | .03 | .54 |
|  | NFA7 | 1.10 | 0.05 | .82 | .03 | .67 |
|  | NFA10 | 0.86 | 0.05 | .64 | .03 | .41 |
| Error correlations | | | | | | |
|  | GStatMost*~  GStatEasy* | 0.21 | 0.03 | .45 | .05 | .21 |
|  | CMQ1~  CMQ2 | 0.26 | 0.03 | .45 | .04 | .20 |
| Factor variances | | | | | | |
| Format preference |  | .29 | .04 | 1.00 | - | - |
| Trust in health authorities |  | .87 | .03 | 1.00 | - | - |
| Conspiracy mentality |  | .37 | .04 | 1.00 | - | - |
| Emotion approach |  | .20 | .04 | 1.00 | - | - |
| Emotion avoidance |  | .56 | .04 | 1.00 | - | - |
|  |  |  |  |  |  |  |
| *Note.* *Reverse coded item. | | | | | | |

| **S8 Table** | | | | | | | | | | | | | |
| --- | --- | --- | --- | --- | --- | --- | --- | --- | --- | --- | --- | --- | --- |
| *Factor Loadings, Variances and Error for the CFA Models – Study 3* | | | | | | | | | | | | | |
|  | |  | | Unstandardized | | | | | Standardized | | | | |
| Latent variable | Item | | | Estimate | | SE | | Estimate | | | SE | | *R*^2^ |
| Factor loadings | | | | | | | | | | | | | |
| Format preference | GStatMost* | | | 1.00 | | - | | .57 | | | .04 | | .33 |
|  | GExpFH | | | 1.51 | | .11 | | .87 | | | .02 | | .75 |
|  | GExpEasy | | | 1.58 | | .11 | | .91 | | | .01 | | .82 |
|  | GExpInf | | | 1.34 | | .10 | | .77 | | | .03 | | .59 |
|  | GStatCont | | | 1.59 | | .11 | | .91 | | | .02 | | .83 |
|  | GStatEasy* | | | 1.45 | | .10 | | .83 | | | .02 | | .69 |
| Trust in health authorities | TrustInfo | | | 1.00 | | - | | .98 | | | .01 | | .95 |
|  | TrustRecommend | | | 0.96 | | .01 | | .94 | | | .01 | | .88 |
|  | TrustOther* | | | 0.73 | | .03 | | .71 | | | .03 | | .51 |
|  | TrustIntent | | | 0.93 | | .02 | | .91 | | | .01 | | .83 |
| Conspiracy mentality | CMQ1 | | | 1.00 | | - | | .59 | | | .04 | | .35 |
|  | CMQ2 | | | 1.13 | | .07 | | .67 | | | .04 | | .44 |
|  | CMQ3 | | | 1.20 | | .09 | | .71 | | | .03 | | .50 |
|  | CMQ4 | | | 1.45 | | .10 | | .86 | | | .02 | | .73 |
|  | CMQ5 | | | 1.40 | | .10 | | .83 | | | .03 | | .68 |
| Emotion approach | NFA2 | | | 1.00 | | - | | .38 | | | .06 | | .15 |
|  | NFA3 | | | 1.52 | | .25 | | .58 | | | .04 | | .34 |
|  | NFA5 | | | 2.04 | | .31 | | .78 | | | .03 | | .61 |
|  | NFA8 | | | 2.32 | | .35 | | .89 | | | .03 | | .79 |
|  | NFA9 | | | 1.51 | | .24 | | .58 | | | .04 | | .34 |
| Emotion avoidance | NFA1 | | | 1.00 | | - | | .71 | | | .03 | | .50 |
|  | NFA4 | | | 1.10 | | .06 | | .78 | | | .03 | | .60 |
|  | NFA6 | | | 0.91 | | .06 | | .65 | | | .04 | | .42 |
|  | NFA7 | | | 1.16 | | .06 | | .82 | | | .03 | | .68 |
|  | NFA10 | | | 1.04 | | .06 | | .74 | | | .03 | | .54 |
| Error correlations | | | | | | | | | | | | | |
|  | GStatMost*~  GStatEasy* | | | .21 | | .03 | | .47 | | | .05 | | .22 |
|  | CMQ1~  CMQ2 | | | .23 | | .04 | | .39 | | | .06 | | .15 |
| Factor variances | | | | | | | | | | | | | |
| Format preference |  | | | .33 | | .05 | | 1.00 | | | - | | - |
| Trust in health authorities |  | | | .95 | | .01 | | 1.00 | | | - | | - |
| Conspiracy mentality |  | | | .35 | | .05 | | 1.00 | | | - | | - |
| Emotion approach |  | | | .15 | | .04 | | 1.00 | | | - | | - |
| Emotion avoidance |  | | | .50 | | .05 | | 1.00 | | | - | | - |
|  | |  |  | |  | |  | | |  | |  | |
| *Note.* *Reverse coded item. | | | | | | | | | | | | | |

In the second sample, the fit indices for the format preference model showed good fit, with all factor loadings being above .50. The fit indices for the trust in health authorities and the emotion approach models showed excellent fit, with all factor loadings being above .30. The fit indices for the conspiracy mentality and emotion avoidance models showed otherwise excellent fit, with the exception of RMSEA, and all factor loadings were above .50. The Cronbach’s alphas were good for all measures: format preference (Cronbach’s α = .90), trust in health authorities (Cronbach’s α = .92), conspiracy mentality (Cronbach’s α = .84), emotion approach (Cronbach’s α = .73), and emotion avoidance (Cronbach’s α = .83).

**Associations Between the Format preference and Theoretically Associated Constructs.** In the first sample, a stronger preference for anecdotes was associated with lower trust in vaccines, *r* = -.50, *p* < .001, lower trust in health authorities, *r* = -.58, *p* < .001, and stronger conspiracy mentality, *r* = .46, *p* < .001. The format preference score was not associated with emotion avoidance, *r* = .08, *p* = .098, emotion approach, *r* = -.04, *p* = .491, or numeracy, *W* = 18254, *p* = .126.

Also in the second sample, higher format preference scores were associated with lower trust in vaccines, *r* = -.66, *p* < .001, lower trust in health authorities, *r* = -.69, *p* < .001, and stronger conspiracy mentality, *r* = .58, *p* < .001. Also here, the format preference score was not associated with emotion avoidance, *r* = .07, *p* = .241, or emotion approach, *r* = .02, *p* = .680. Finally, the median format preference score was lower for participants’ high in numeracy, Mdn = 2, MAD = 0.99, compared to participants low in numeracy, Mdn = 2.17, MAD = 1.24, *W* = 10400, *p* = .033.

**Does Format Preference Predict Susceptibility to Anecdotal Information?** The pre- and post-anecdote mean scores and the mean change scores for both samples are presented in Tables S9 and S10. See Tables S11 and S12 for the multiple regression models.

| **S9 Table** | | | | | | |
| --- | --- | --- | --- | --- | --- | --- |
| *Vaccination Intention and Attitude Mean Scores by Anecdote Group – Study 2* | | | | | | |
| Variable | Pro-vaccine | | Anti-vaccine | | Control | |
|  | Mean | SD | Mean | SD | Mean | SD |
| Pre-manipulation | | | | | | |
| Vaccination intention | 68.99 | 24.85 | 64.45 | 27.79 | 67.01 | 26.04 |
| Perceived disease threat | 54.73 | 21.04 | 51.86 | 24.61 | 51.21 | 21.07 |
| Perceived vaccine safety | 76.83 | 23.56 | 75.20 | 25.62 | 77.85 | 22.70 |
| Perceived vaccine efficacy | 66.50 | 18.91 | 65.13 | 22.91 | 65.16 | 18.95 |
| Post-manipulation | | | | | | |
| Vaccination intention | 67.91 | 25.55 | 64.10 | 28.46 | 65.02 | 26.26 |
| Perceived disease threat | 56.48 | 21.97 | 52.99 | 25.35 | 51.66 | 22.98 |
| Perceived vaccine safety | 76.79 | 24.44 | 72.99 | 26.91 | 76.32 | 23.46 |
| Perceived vaccine efficacy | 69.52 | 20.77 | 64.72 | 24.01 | 65.35 | 19.82 |
| Change score | | | | | | |
| Vaccination intention | -1.08 | 7.26 | -0.35 | 6.31 | -1.99 | 5.38 |
| Perceived disease threat | 1.74 | 7.72 | 1.13 | 5.80 | 0.45 | 6.70 |
| Perceived vaccine safety | -0.04 | 7.64 | -2.22 | -0.17 | -1.54 | 5.16 |
| Perceived vaccine efficacy | 3.02 | 8.78 | -0.41 | 6.04 | 0.19 | 4.39 |
| *Note.* Pro-vaccine = group that received pro-vaccine anecdotes, Anti-vaccine = group that received anti-vaccine anecdotes, Control = group that received material unrelated to vaccines. | | | | | | |

| **S10 Table** | | | | | | |
| --- | --- | --- | --- | --- | --- | --- |
| *Vaccination Intention and Attitude Mean Scores by Anecdote Type – Study 3* | | | | | | |
| Variable | Control | | Anti-vaccine | | Pro-vaccine | |
|  | Mean | SD | Mean | SD | Mean | SD |
| Pre-manipulation | | | | | | |
| Vaccination intention | 64.63 | 34.41 | 60.48 | 36.04 | 60.42 | 36.09 |
| Perceived disease threat | 51.74 | 30.53 | 47.94 | 32.31 | 47.80 | 31.74 |
| Perceived vaccine safety | 72.18 | 27.69 | 71.29 | 27.91 | 73.67 | 28.53 |
| Perceived vaccine efficacy | 63.45 | 25.86 | 63.11 | 27.33 | 64.06 | 26.25 |
| Post-manipulation | | | | | | |
| Vaccination intention | 62.98 | 34.21 | 57.71 | 35.37 | 60.10 | 36.07 |
| Perceived disease threat | 52.36 | 31.05 | 48.88 | 32.55 | 49.82 | 32.82 |
| Perceived vaccine safety | 70.42 | 27.94 | 68.60 | 29.71 | 73.08 | 28.33 |
| Perceived vaccine efficacy | 64.24 | 26.12 | 60.47 | 28.68 | 66.19 | 26.83 |
| Change score | | | | | | |
| Vaccination intention | -1.64 | 8.69 | -2.77 | 12.33 | -0.31 | 10.63 |
| Perceived disease threat | 0.62 | 9.23 | 0.94 | 12.28 | 2.02 | 12.65 |
| Perceived vaccine safety | -1.76 | 8.19 | -2.69 | 13.06 | -0.58 | 9.00 |
| Perceived vaccine efficacy | 0.80 | 7.53 | -2.64 | 13.92 | 2.13 | 9.71 |
| *Note.* Pro-vaccine = pro-vaccine anecdotes, Anti-vaccine = anti-vaccine anecdotes, Control = anecdotes unrelated to vaccines. | | | | | | |

| **S11 Table** | | | | | | | | | | | | | | | |
| --- | --- | --- | --- | --- | --- | --- | --- | --- | --- | --- | --- | --- | --- | --- | --- |
| *Multiple Regression Model – Study 2* | | | | | | | | | | | | | | | |
| Variable | Vaccination intention | | |  | Disease threat | | |  | Vaccine safety | | |  | Vaccine efficacy | | |
|  | *b* | 95% CI | *p* |  | *b* | 95% CI | *p* |  | *b* | 95% CI | *p* |  | *b* | 95% CI | *p* |
| Intercept | -2.01 | (-3.10, -0.92) | .000 |  | 0.35 | (-0.83, 1.53) | .563 |  | -1.49 | (-2.62, -0.36) | .010 |  | 0.15 | (-1.00, 1.29) | .802 |
| FormPref | -0.19 | (-1.35, 0.97) | .746 |  | -0.96 | (-2.22, 0.30) | .134 |  | 0.36 | (-0.84, 1.57) | .553 |  | -0.55 | (-1.78, 0.67) | .375 |
| Pro-vaccine | 0.87 | (-0.63, 2.38) | .255 |  | 1.40 | (-0.23, 3.03) | .093 |  | 1.42 | (-0.14, 2.98) | .075 |  | 2.85 | (1.26, 4.44) | .000 |
| Anti-vaccine | 1.68 | (0.05, 3.31) | .044 |  | 0.69 | (-1.08, 2.46) | .441 |  | -0.73 | (-2.42, 0.97) | .399 |  | -0.61 | (-2.33, 1.11) | .485 |
| FormPref*Pro-vaccine | -0.87 | (-2.47, 0.73) | .284 |  | 0.87 | (-0.84, 2.59) | .318 |  | 0.39 | (-1.25, 2.03) | .643 |  | 1.06 | (-0.61, 2.73) | .214 |
| FormPref*Anti-vaccine | 1.57 | (-0.02, 3.15) | .052 |  | 1.18 | (-0.55, 2.91) | .181 |  | -1.94 | (-3.60, -0.29) | .022 |  | -0.94 | (-2.62, 0.75) | .276 |
| *Note.* FormPref = format preference, Pro-vaccine = Pro-vaccine group, Anti-vaccine = Anti-vaccine group. | | | | | | | | | | | | | | | |

| **S12 Table** | | | | | | | | | | | | | | | |
| --- | --- | --- | --- | --- | --- | --- | --- | --- | --- | --- | --- | --- | --- | --- | --- |
| Multiple Regression Model – Preparation Study 3 | | | | | | | | | | | | | | | |
| Variable | Vaccination intention | | |  | Disease threat | | |  | Vaccine safety | | |  | Vaccine efficacy | | |
|  | *b* | 95% CI | *p* |  | *b* | 95% CI | *p* |  | *b* | 95% CI | *p* |  | *b* | 95% CI | *p* |
| Intercept | -1.65 | (-2.87, -0.43) | .008 |  | 0.63 | (-0.69, 1.95) | .348 |  | -1.81 | (-2.97, -0.65) | .002 |  | 0.74 | (-0.46, 1.95) | .224 |
| FormPref | -0.04 | (-1.27, 1.18) | .944 |  | -0.84 | (-2.15, 0.48) | .214 |  | -0.66 | (-1.82, 0.50) | .263 |  | -0.42 | (-1.62, 0.78) | .494 |
| Pro-vaccine | 1.33 | (-0.37, 3.03) | .125 |  | 1.40 | (-0.40, 3.21) | .128 |  | 1.16 | (-0.48, 2.80) | .165 |  | 1.36 | (-0.34, 3.06) | .116 |
| Anti-vaccine | -0.97 | (-2.67, 0.73) | .264 |  | 0.56 | (-1.24, 2.37) | .542 |  | -0.77 | (-2.41, 0.87) | .357 |  | -3.15 | (-4.85, -1.45) | .000 |
| FormPref*Pro-vaccine | 1.22 | (-0.48, 2.92) | .159 |  | 0.50 | (-1.31, 2.30) | .590 |  | 2.11 | (0.47, 3.75) | .012 |  | 1.85 | (0.15, 3.55) | .033 |
| FormPref*Anti-vaccine | -2.62 | (-4.33, -0.92) | .003 |  | -1.82 | (-3.63, -0.02) | .048 |  | -3.18 | (-4.82, -1.54) | .000 |  | -3.90 | (-5.60, -2.20) | .000 |
| *Note.* FormPref = format preference, Pro-vaccine = Pro-vaccine anecdotes, Anti-vaccine = Anti-vaccine anecdotes. | | | | | | | | | | | | | | | |

***Vaccination Intention.*** In the first sample, the interaction between format preference and the Pro-vaccine group was not statistically significant, demonstrating that the effect of format preference on vaccination intentions was of similar size in the Pro-vaccine group and the Control group. The interaction between format preference and the Anti-vaccine group was also not statistically significant, indicating that the effect of format preference on vaccination intentions was of similar size in the Anti-vaccine group and the Control group. However, in the second sample, the interaction was statistically significant between format preference and the anti-vaccine anecdotes, indicating that the effect of format preference was different for the anti-vaccine anecdotes relative to the control anecdotes. The simple effect of format preference on vaccination intentions for the anti-vaccine anecdotes was negative and statistically significant, *b* = -2.67, CI[-3.89, -1.45], *p* < .001, meaning that the more a person preferred anecdotes the more their vaccination intentions were negatively affected by the anti-vaccine anecdotes. The interaction was not statistically significant between format preference and the pro-vaccine anecdotes, suggesting that the effect of format preference on vaccination intentions was of similar size for the pro-vaccine anecdotes and the control anecdotes.

***Perceived Disease Threat.*** In the first sample, the interaction between format preference and the Pro-vaccine group was not statistically significant, suggesting that the effect of format preference on perceived disease threat did not differ between the Pro-vaccine group and the Control group. Similarly, the interaction between format preference and the Anti-vaccine group was not statistically significant, indicating that the effect of format preference on perceived disease threat was similar between the Anti-vaccine group and the Control group. However, in the second sample, the interaction was statistically significant between format preference and the anti-vaccine anecdotes, suggesting that the effect of format preference on perceived disease threat was different for the anti-vaccine anecdotes compared to the control anecdotes. The simple effect of format preference on perceived disease threat for the anti-vaccine anecdotes was negative and statistically significant, *b* = -2.66, CI[-3.98, -1.34], *p* < .001, meaning that the more a person preferred anecdotes the more their disease threat perceptions were negatively affected by the anti-vaccine anecdotes. The interaction between format preference and the pro-vaccine anecdotes was not statistically significant, demonstrating that the effect of format preference on perceived disease threat was of similar size for the pro-vaccine anecdotes and the control anecdotes.

***Perceived Vaccine Safety.*** In the first sample, the interaction between format preference and the Anti-vaccine group was statistically significant, demonstrating that the effect of format preference was different in the Anti-vaccine group compared to the Control group. The simple effect of format preference on perceived vaccine safety in the Anti-vaccine group was negative and statistically significant, *b* = -1.58, CI[-2.72, -0.44], *p* = .007, meaning that the more a person preferred anecdotes the more negatively they were affected by the anti-vaccine anecdotes. The interaction between format preference and the Pro-vaccine group was, however, not statistically significant, indicating that the effect of format preference on perceived vaccine safety did not differ between the Pro-vaccine group and the Control group. In the second sample, the interaction between format preference and the anti-vaccine anecdotes was statistically significant, meaning that the effect of format preference on perceived vaccine safety was different for the anti-vaccine anecdotes in relation to the control anecdotes. The simple effect of format preference on perceived vaccine safety was statistically significant for the anti-vaccine anecdotes, so that the anti-vaccine anecdotes had a greater negative effect, *b* = -3.84, CI[-5.00, -2.68], *p* < .001, on perceived vaccine safety, the more a person preferred anecdotes. The interaction between format preference and the pro-vaccine anecdotes was also statistically significant, suggesting that the effect of format preference for the pro-vaccine anecdotes differed from the control anecdotes. The simple effect of format preference on perceived vaccine safety was statistically significant for the pro-vaccine anecdotes, so that the pro-vaccine anecdotes had a greater positive effect, *b* = 1.45, CI[0.29, 2.61], *p* =.014, on perceived vaccine safety, the more a person preferred anecdotes.

***Perceived Vaccine Efficacy.*** In the first sample, the interaction between format preference and the Pro-vaccine group was not statistically significant, suggesting that the effect of format preference on perceived vaccine efficacy was equally small in the Control group and the Pro-vaccine group. The interaction between format preference and the Anti-vaccine group was also not statistically significant, meaning that the effect of format preference on perceived vaccine efficacy did not differ between the Control group and the Anti-vaccine group. In the second sample, the interaction between format preference and the anti-vaccine anecdotes was statistically significant, demonstrating that the effect of format preference for the anti-vaccine anecdotes was different relative to the control anecdotes. The simple effect of format preference on perceived vaccine efficacy was statistically significant for the anti-vaccine anecdotes, so again that the anti-vaccine anecdotes had a greater negative effect, *b* = -4.32, CI[-5.52 – -3.12], *p* < .001, on the perceived vaccine efficacy, the more a person preferred anecdotes. Lastly, the interaction between format preference and the pro-vaccine anecdotes was statistically significant, indicating that the effect of format preference on perceived vaccine efficacy for the pro-vaccine anecdotes and control anecdotes differed from each other. The simple effect of format preference on perceived vaccine efficacy was statistically significant for the pro-vaccine anecdotes, so that the pro-vaccine anecdotes had a greater positive effect on the perceived vaccine efficacy, the more a person preferred anecdotes, *b* = 1.43, CI[0.23 – 2.63], *p* =.020.

***Discussion of the Scale Validation***

On the whole, the format preference scale demonstrated good scale reliability and was associated with most of the theoretically related factors in the expected way. The results from the analyses investigating whether people’s preference for anecdotes is associated with how susceptible their vaccination attitudes and intentions are to pro- and anti-vaccine anecdotes were inconsistent. However, in general, participants who reported stronger format preference were more affected by both pro- and anti-vaccine anecdotes. The association was stronger after reading anti-vaccine anecdotes than pro-vaccine anecdotes. It is possible that the lack of statistically significant relationships between the format preference scale and most of the measures of susceptibility to anecdotal information was due to characteristics of the experimental task. One such example is that the anecdotal manipulation was not efficient enough to elicit a change in all the outcome variables or that the outcome variables were not sensitive enough to detect a change.

Taken together, these three studies indicate that the format preference scale can be used as an indication of how much a person will change their vaccine attitudes and intentions after reading anecdotes. We will thus proceed to investigate whether tailoring COVID-19 and influenza vaccine hesitancy interventions by intervention style (statistical vs anecdotal) leads to better intervention outcomes.

**References**

Appel, M., Gnambs, T., & Maio, G. R. (2012). A Short Measure of the Need for Affect. *Journal of Personality Assessment*, *94*(4), 418–426. https://doi.org/10.1080/00223891.2012.666921

Betsch, C., Schmid, P., Heinemeier, D., Korn, L., Holtmann, C., & Böhm, R. (2018). Beyond confidence: Development of a measure assessing the 5C psychological antecedents of vaccination. *PLOS ONE*, *13*(12), e0208601. https://doi.org/10.1371/journal.pone.0208601

Betsch, C., Ulshöfer, C., Renkewitz, F., & Betsch, T. (2011). The Influence of Narrative v. Statistical Information on Perceiving Vaccination Risks. *Medical Decision Making*, *31*(5), 742–753. https://doi.org/10.1177/0272989X11400419

Bruder, M., Haffke, P., Neave, N., Nouripanah, N., & Imhoff, R. (2013). Measuring Individual Differences in Generic Beliefs in Conspiracy Theories Across Cultures: Conspiracy Mentality Questionnaire. *Frontiers in Psychology*, *4*. https://doi.org/10.3389/fpsyg.2013.00225

Cokely, E., Galesic, M., Schulz, E., Ghazal, S., & Garcia-Retamero, R. (2012). Measuring Risk Literacy: The Berlin Numeracy Test. *Judgment and Decision Making*, *7*. https://doi.org/10.1037/t45862-000

Karlsson, L. C., Lewandowsky, S., Antfolk, J., Salo, P., Lindfelt, M., Oksanen, T., Kivimäki, M., & Soveri, A. (2019). The association between vaccination confidence, vaccination behavior, and willingness to recommend vaccines among Finnish healthcare workers. *PLOS ONE*, *14*(10), e0224330. https://doi.org/10.1371/journal.pone.0224330
